# Improved Immunotherapy Outcomes via Cuproptosis Upregulation of HLA-DRA Expression: Promoting the Aggregation of CD4^+^ and CD8^+^T Lymphocytes in Clear Cell Renal Cell Carcinoma

**DOI:** 10.3390/ph17060678

**Published:** 2024-05-24

**Authors:** Bowen Wang, Yiwen Liu, Feng Xiong, Chunyang Wang

**Affiliations:** Urology Surgery Department, The First Affiliated Hospital of Harbin Medical University, Youzheng Street #37, Nangang District, Harbin 150001, China

**Keywords:** cuproptosis, human leukocyte antigen, tumor-specific major histocompatibility complex-II-II, clear cell renal cell carcinoma, immunotherapy, immune checkpoint inhibitors

## Abstract

Immunotherapy has shown promising clinical results in clear cell renal cell carcinoma (ccRCC), but low clinical target response rates due to dysfunction of the major histocompatibility complex (MHC) and an inhibitory tumor immune microenvironment (TIME) have largely limited the associated clinical benefits. In the present study, we explored the feasibility of enhancing tumor-specific-MHC-II-HLA-DRA expression, counteracting the TIME’s suppressive effects, thereby improving the sensitivity of immune checkpoint inhibitor (ICI) therapy from the standpoint of cuproptosis. Immunohistochemical staining and in vitro experiments validated the expression of HLA-DRA in ccRCC and its positive impact on ICI therapy. Subsequently, we observed that cuproptosis upregulated HLA-DRA expression in a dose-dependent manner, further confirming the link between cuproptosis and HLA-DRA. In vivo experiments showed that cuproptosis increased the sensitivity to ICI treatment, and implementing cuproptosis alongside anti-PD-1 treatment curtailed tumor growth. Mechanistically, cuproptosis upregulates HLA-DRA expression at the transcriptional level in a dose-dependent manner by inducing the production of reactive oxygen species; high levels of HLA-DRA promote the expression of chemokines CCL5, CXCL9, and CXCL10 in the TIME, inhibiting the development of a pro-tumor microenvironment by promoting the infiltration of CD4^+^T and CD8^+^T cells, thereby synergizing ICI therapy and exerting anti-tumor effects. Taken together, this work highlights the role of cuproptosis in mediating TIME remodeling and synergistic immunotherapy, providing new evidence that cuproptosis can evoke effective anti-tumor immune responses.

## 1. Introduction

Renal cell carcinoma (RCC) is one of the most common malignancies of the urinary system [1]. Approximately 75–80% of RCC cases are clear cell renal cell carcinoma (ccRCC), according to the pathological classification [2]. Globally, the 5-year survival rate of patients diagnosed with metastatic or advanced kidney cancer is only 12.1% [3,4,5]. With the development of a series of anti-human programmed death-1 (PD-1)/programmed death ligand-1 (PD-L1) monoclonal antibodies (pembrolizumab/attuzumab) and other immune checkpoint inhibitors (ICIs), the overall survival (OS) of some patients with stage III/IV renal cancer has greatly improved [6,7], ushering in a new era of modern oncology. However, low clinical target response rates and ICI resistance caused by dysfunction of the major histocompatibility complex (MHC) and immunosuppressive tumor microenvironment (TME) limit their potential utility [8,9,10]. At present, the combined effectiveness rate of ICI therapy is less than 20% [11], making it crucial to improve the therapeutic responsiveness of ICIs [12]. In this study, the researchers aimed to improve the expression of MHC molecules in tumors and gain a deeper understanding of the TME. The ultimate goal is to develop effective therapeutic strategies to enhance the effects of immunotherapy in patients with ccRCC.

Cuproptosis is a recently discovered form of regulated cell death (RCD), which has been shown to effectively inhibit tumor growth [13,14,15,16]. Copper ions influence tumor growth by participating in immune cells’ survival, differentiation, activation, transit, and function formation [17,18,19]. This suggests that cuproptosis is of great biological potential and is a promising area of research for regulating the TME and anti-tumor immune responses.

Human leukocyte antigen-DRA (HLA-DRA) is mainly involved in the encoding of the MHC-II alpha chain. Previously, it was believed that MHC-II molecules were mainly expressed by antigen-presenting cells (APCs) [20]. However, recent studies have shown that tumor cells also express MHC-II molecules, known as tumor-specific major histocompatibility complex II (ts-MHC-II) [21,22,23]. The expression of ts-MHC-II enhances tumor recognition by the immune system. For example, in patients with triple-negative breast cancer (TNBC), the aberrant expression of the MHC-II pathway in tumor cells triggers an anti-tumor immune response, which reduces the rate of recurrence and improves progression-free survival (PFS) [24]. Moreover, MHC-II expression is strongly associated with an increase in the number of tumor-infiltrating lymphocytes (TILs) and interferon signaling in TNBC [25]. The expression of ts-MHC-II in melanoma cells was found to be correlated with IFN-γ and IFN-γ-mediated gene signatures, and it was also found to predict anti-PD-1 responses [26]. Similar results were observed in patients with relapsed/refractory classical Hodgkin’s lymphoma who were treated with nivolumab, a PD-1 antibody [27]. Although these findings suggest that MHC-II plays an important role in activating immune responses, antigen presentation, and ICI treatment, studies on ts-MHC-II are scarce, and the existing studies analyzing its role in renal cell carcinoma are limited [28,29]. Therefore, further studies are needed to analyze the possible functional effects of cuproptosis and HLA-DRA, which may provide new ideas for ccRCC immunotherapy.

This article utilizes in-house transcriptome sequencing data, bioinformatics analysis, and both in vivo and in vitro experimental validation to provide a comprehensive description of cuproptosis and the role of ts-MHC-II-HLA-DRA in ccRCC immunotherapy. Our findings indicate that cuproptosis can modulate the expression of HLA-DRA, leading to improved immunotherapeutic sensitivity of ccRCC. Cuproptosis has the potential to reverse the suppressive tumor immune microenvironment of ccRCC by stimulating HLA-DRA expression and modulating immune cells and related immune responses. Additionally, in vivo experiments indicate that cuproptosis could enhance the sensitivity to ICI treatment by recruiting cytotoxic T lymphocytes (CTLs). This study expands our understanding of the regulation of ts-MHC-II expression and provides evidence that cuproptosis and HLA-DRA promote the body’s anti-tumor immune response. Cuproptosis enhances the sensitivity to ICI treatment in vivo, offering new insights into prolonging the survival of patients with advanced renal cancer and improving immunotherapy for ccRCC.

## 2. Results

### 2.1. Key Cuproptosis-Related Gene in ccRCC, HLA-DRA

**Figure 1:**
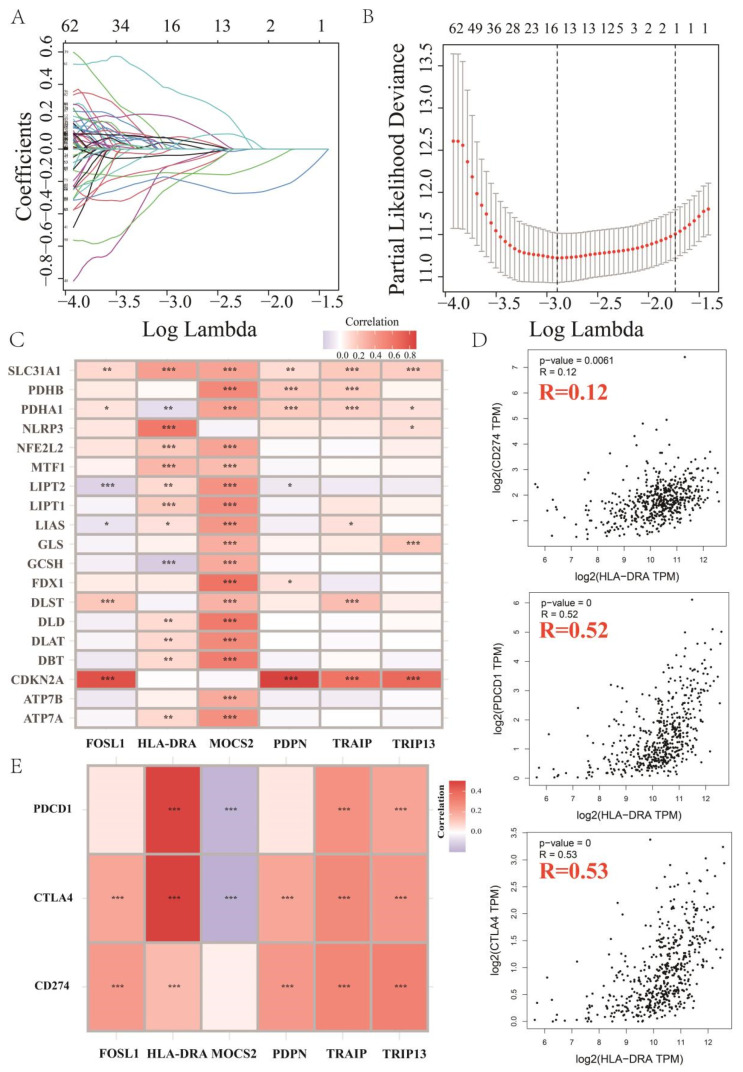
HLA-DRA is a key cuproptosis-related gene in ccRCC. (**A**) LASSO coefficient distribution of 266 CRGs significantly associated with overall survival. (**B**) Random grouping of models for LASSO Cox regression to determine the best lambda value. (**C**) Co-expression heatmap of six CRGs (MOCS2, TRIP13, HLA-DRA, TRAIP, PDPN, and FOSL1) and the 19 cuproptosis genes (NFE2L2, NLRP3, ATP7B, ATP7A, SLC31A1, FDX1, LIAS, LIPT1, LIPT2, DLD, DLAT, PDHA1, PDHB, MTF1, GLS, CDKN2A, DBT, GCSH, and DLST). (**D**) Co-expression of HLA-DRA with three immune checkpoints (PDCD1, CTLA-4, and CD274) in ccRCC samples was analyzed according to the GEPIA database. (**E**) Co-expression of CRGs with PDCD1, CTLA-4, and CD274 in a cohort of 613 ccRCC samples in the TCGA database (* *p* < 0.05, ** *p* < 0.01, *** *p* < 0.001).

Given the important role of cuproptosis in regulating the TME and the anti-tumor immune response, our study aimed to identify the key CRGs, to reveal the pathways through which cuproptosis exerts its biological functions. A total of 16,876 lncRNAs and 19,938 mRNAs were identified in the existing transcripts, along with the expression of related genes, including 19 genes involved in the regulation of cuproptosis (*NFE2L2*, *NLRP3*, *ATP7B*, *ATP7A*, *SLC31A1*, *FDX1*, *LIAS*, *LIPT1*, *LIPT2*, *DLD*, *DLAT*, *PDHA1*, *PDHB*, *MTF1*, *GLS*, *CDKN2A*, *DBT*, *GCSH*, and *DLST*) in patients with ccRCC. Co-expression analysis of mRNAs and 19 key genes (for stringent screening, we set the cor filter > 0.6, *p* < 0.01) resulted in 6899 CRGs and formed a Sankey diagram (Supplementary Figure S1). Data on the expression of CRGs were combined with survival data and were randomly divided into groups. From the 6899 genes, univariate regression analysis was performed to identify 266 CRGs that were significantly associated with OS (*p* < 0.01). Thereafter, LASSO regression analysis was performed on those 266 CRGs (Figure 1A). The results were cross-validated to determine the lambda values with the smallest error (Figure 1B). Finally, multivariate Cox regression analysis was conducted to identify the six optimized CRGs (*MOCS2*, *TRIP13*, *HLA-DRA*, *TRAIP*, *PDPN*, and *FOSL1*). Additionally, we analyzed the co-expression relationships between the six CRGs and the cuproptosis genes (Figure 1C). 

GO enrichment analysis revealed that the CRGs were mainly enriched in the humoral immune response, receptor ligand activity, signaling receptor activator, and signaling receptor activator activity pathways (Supplementary Figure S2A,B). According to the KEGG enrichment analysis, CRGs were mainly enriched in the NF-kappa B, IL-17, PI3K-Akt, drug metabolism-cytochrome P450, and TGF-beta signaling pathways (Supplementary Figure S2C,D). These results provide a foundation for further exploration of the biological processes in which CRGs may be involved. Functional enrichment analyses revealed that CRGs may play crucial roles in tumorigenesis and immune infiltration.

Subsequently, we investigated the correlation between CRGs and immune checkpoints to understand the potential role of CRGs in the anti-tumor process. We examined the correlation of each gene in the CRGs with PD-1, PD-L1, and CTLA4 (Figure 1D; Supplementary Figure S3A–E), and we found that HLA-DRA was positively correlated with the expression of these three immune checkpoints (Figure 1D). To ensure the accuracy of our results, we extracted all 613 ccRCC samples from the TCGA database and determined the expression levels of PD-1 (PDCD1), PD-L1 (CD274), and CTLA4 in each sample. Co-expression analyses of all six CRGs with PD-1, PD-L1, and CTLA4 were performed. Consistent with the above-mentioned results, it was found that HLA-DRA was positively correlated with the expression of all three genes (PD-1, PD-L1, and CTLA4), and the correlation between HLA-DRA, PDCD1, and CTLA4 in terms of co-expression was highly significant among all six CRGs (Figure 1E). These findings suggest that HLA-DRA may play a crucial role in mediating anti-tumor immune responses. 

### 2.2. HLA-DRA Expressed as an MHC-II in ccRCC Cell Lines and Pathological Tissues

**Figure 2:**
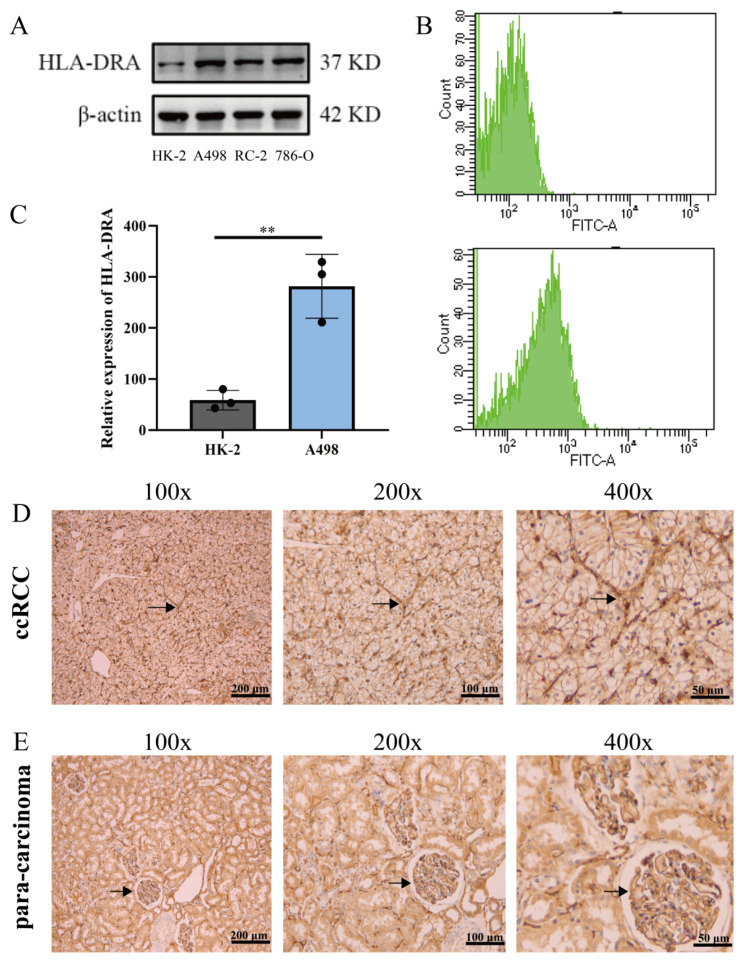
HLA-DRA expressed as an MHC-II in ccRCC. (**A**) Detection of HLA-DRA protein expression in HK-2, A498, RC-2, and 786-O cells through Western blotting (WB). (**B**,**C**) Expression of HLA-DRA in HK-2 and A498 cells was assessed through flow cytometry. (**D**,**E**) HLA-DRA expression in ccRCC and peritumoral tissues. (The black arrows indicated examples of HLA-DRA expression in tissues), (** *p* < 0.01).

It is important to note that HLA-DRA is an MHC-II molecule. Previously, it was thought that MHC-II molecules were expressed only in antigen-presenting cells [30]. However, recent studies have shown that a variety of tumors can also express tumor-specific MHC-II molecules and play an important role in their function [21]. Nevertheless, studies on HLA-DRA in kidney cancer are limited and have mainly focused on bioinformatics analysis. We analyzed the expression of HLA-DRA in ccRCC cells and renal tubular epithelial cells (HK-2) through Western blotting (Figure 2A) and flow cytometry detection (Figure 2B,C). The results showed that HLA-DRA was expressed in both ccRCC cells and HK-2 cells, with higher expression levels in ccRCC cells. Immunohistochemical (IHC) staining was used to analyze the expression of HLA-DRA in renal carcinoma and paracancerous tissues. The results demonstrated that HLA-DRA was expressed in both renal carcinoma and peritumoral tissues (Figure 2D,E). The experiments above verified the expression of HLA-DRA as an MHC II molecule in ccRCC.

### 2.3. High HLA-DRA Expression Enhances the Sensitivity of ccRCC to ICI Treatment

**Figure 3:**
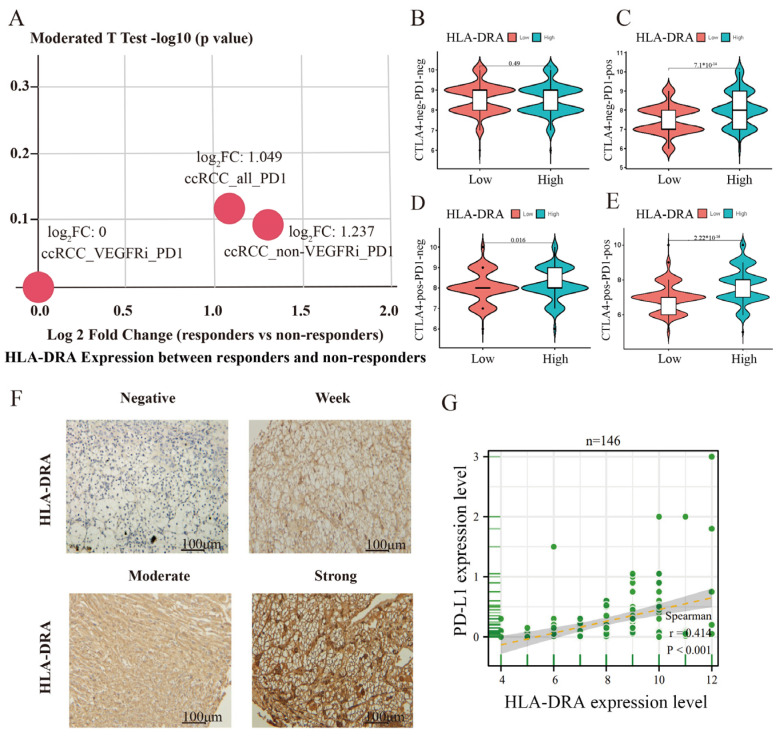
High HLA-DRA expression predicted greater responsiveness to ICI therapy. (**A**) Differences in HLA-DRA expression between twelve patients who responded and those who did not respond to anti-PD-1 treatment in ccRCC were analyzed using the “immunotherapy” module in the TISIDB database. (**B**–**E**) The impact of HLA-DRA expression on the treatment outcomes of patients who had received no immunotherapy (anti-PD-1 or anti-CTLA-4)/anti-PD-1/anti-CTLA-4/anti-CTLA4 combined with anti-PD-1 treatment were analyzed using R software (4.4.0). (**F**) Representative immunohistochemical images of HLA-DRA protein expression in a ccRCC tissue microarray from 146 patients. (**G**) Spearman’s correlation analysis of HLA-DRA and PD-L1 protein expression in 146 ccRCC tissue samples.

As tsMHC-II is linked to predicting the responsiveness to ICI therapy, we investigated whether HLA-DRA expression affects the sensitivity to anti-CTLA4 or anti-PD-1/PD-L1 treatment. We compared HLA-DRA expression between anti-PD1-responsive and -non-responsive patients (the patient’s anti-PD-1 therapeutic agent was a nivolumab monoclonal antibody) in the TISIDB database. This study’s findings indicate that HLA-DRA expression has an impact on the response to nivolumab in ccRCC patients. Specifically, patients with higher HLA-DRA expression exhibited a better response to nivolumab treatment (Figure 3A; Table S1). HLA-DRA expression did not influence the therapeutic effect in patients who did not receive CTLA4 and PD-1 monoclonal antibodies (*p* = 0.49) (Figure 3B). However, patients treated with PD-1 mAb alone (*p* = 7.1 × 10^−14^) (Figure 3C) or CTLA4 mAb alone (*p* = 0.016) (Figure 3D) showed stronger treatment effects when HLA-DRA expression was high. Furthermore, the combination of CTLA-4 mAb and PD-1 mAb also had a better effect on patients with high HLA-DRA expression (Figure 3E). These results suggest a positive correlation between HLA-DRA expression and the effectiveness of immunotherapy in ccRCC patients. To ensure the reliability of our conclusions, we conducted IHC testing for HLA-DRA expression on 150 ccRCC tissues using an independent in-house dataset (4 out of 150 cases were not included in the statistics due to a missing IHC testing process) (Figure 3F). Given that the expression level of PD-L1 can be used as a predictor of a patient’s sensitivity to ICI therapy, we examined PD-L1 protein expression in 146 ccRCC tissue samples and performed Pearson’s correlation analysis between HLA-DRA and PD-L1 expression. Our findings revealed a positive correlation between HLA-DRA and PD-L1 expression (Figure 3G). In conclusion, we have validated the predictive value of HLA-DRA in determining the responsiveness of cancer patients to ICI therapy.

### 2.4. HLA-DRA Positively Affects the Immune Microenvironment of ccRCC

**Figure 4:**
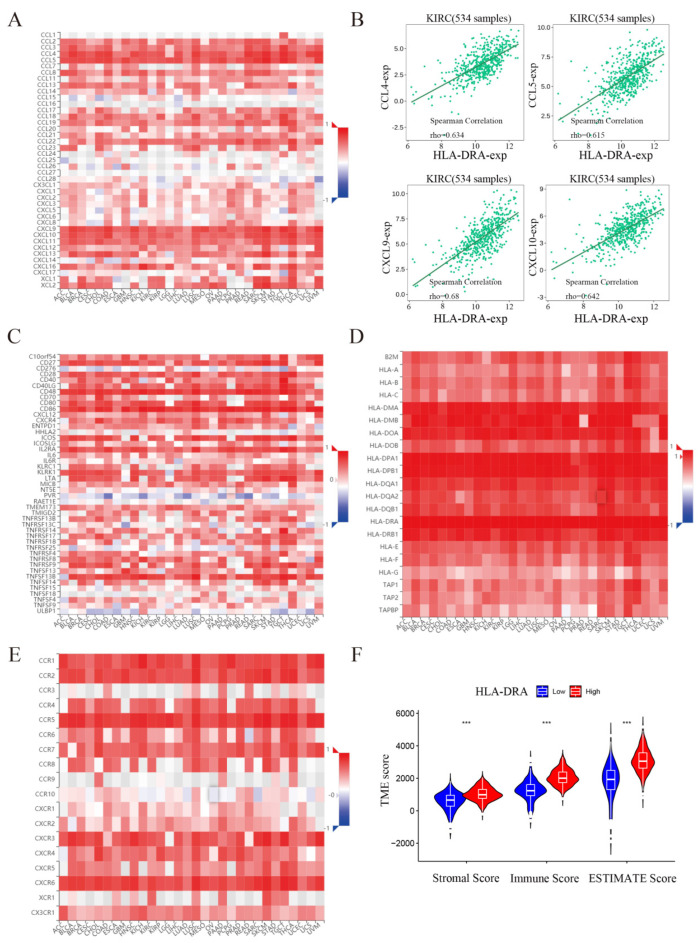
HLA−DRA has a positive impact on the immune microenvironment in ccRCC. (**A**) Correlation analysis of HLA-DRA and chemokines using the TISIDB database. The horizontal coordinates represent the corresponding abbreviations for different cancer types. (**B**) Correlation analysis of HLA-DRA with CCL4, CCL5, CXCL9, and CXCL10 in 534 KIRC patients was performed by Spearman’s analysis in the TISIDB database. (**C**) Correlation analysis of HLA-DRA and immunostimulants using the TISIDB database. The horizontal coordinates represent the corresponding abbreviations for different cancer types. (**D**) Correlation analysis of HLA-DRA and MHC molecules using the TISIDB database. The horizontal coordinates represent the corresponding abbreviations for different cancer types. (**E**) Correlation analysis of HLA-DRA and chemokine receptors using the TISIDB database. The horizontal coordinates represent the corresponding abbreviations for different cancer types. (**F**) Comparison of TME scores between high and low HLA-DRA expression groups using the R software (*** *p* < 0.001) (the abbreviation KIRC denotes clear cell renal cell carcinoma).

To investigate the effect of HLA-DRA on ICI treatment, we conducted a single-gene GSEA enrichment analysis. Our findings indicated that HLA-DRA is enriched in antigen processing and presentation, as well as in the T-cell receptor, toll-like receptor, and B-cell receptor signaling pathways (Supplementary Figure S4). We conducted a series of comprehensive analyses to determine the possible functions of HLA-DRA in the immune system. In RCC, HLA-DRA was found to be closely associated with most chemokines (Figure 4A). HLA-DRA also showed a surprising positive correlation with CCL4, CCL5, CXCL9, and CXCL10 (Figure 4B), indicating its beneficial role in the human immune response through inducing immune cell infiltration and improving tumor clearance in the body. Further analysis showed that HLA-DRA is positively correlated with most immunostimulatory molecules (Figure 4C), MHC molecules (Figure 4D), and chemokine receptors (Figure 4E). These findings suggest that HLA-DRA plays an active role in the ccRCC TME. Additional analysis of immune cell infiltration (Supplementary Figure S5A,B) revealed that the HLA-DRA high-expression group had increased levels of CD8^+^ T cells, T-follicular helper cells, M1 and M2 macrophages, resting dendritic cells, eosinophils, and neutrophils, while the low-expression group had decreased levels of B cells, resting natural killer (NK) cells, M0 macrophages, and resting mast cells.

As the two primary non-tumor components of the TME, immune cells and stromal cells play a crucial role in tumor prognosis. To determine the TME of the HLA-DRA high- and low-expression groups, we calculated the ESTIMATE scores and estimated the purity of stromal, immune, and tumor cells. Our findings indicated that the stromal, immune, and ESTIMATE scores were significantly higher in the HLA-DRA high-expression group than in the low-expression group (Figure 4F). Given the widely recognized impact of the TME on the body’s immunotherapeutic response, HLA-DRA may enhance the therapeutic sensitivity of ICIs by improving the TME.

### 2.5. Cuproptosis Upregulates the Protein Expression of HLA-DRA from the Transcriptional Level in a Dose-Dependent Manner through Reactive Oxygen Species

**Figure 5:**
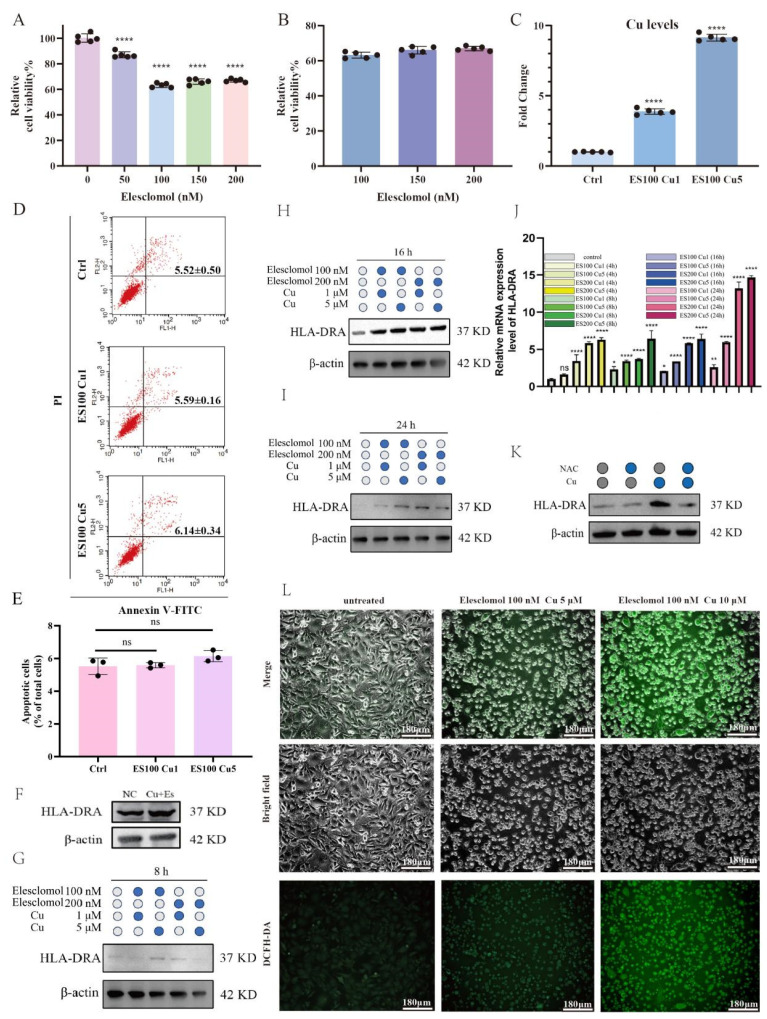
Cuproptosis upregulates HLA-DRA protein expression level from the transcriptional level in a dose-dependent manner via reactive oxygen species. (**A**,**B**) The viability of A498 cells was evaluated via CCK8 assay after being exposed to various concentrations of elesclomol. (**C**) Intracellular copper ion levels were measured after treatment with elesclomol (100 nM) and Cu (1 µM, 5 µM). (**D**,**E**) Levels of apoptosis were analyzed after treatment with elesclomol (100 nM) and Cu (1 µM, 5 µM) through flow cytometry detection. (**F**) HLA-DRA expression levels in A498 cells were measured through WB after treatment with elesclomol (100 nM) and Cu (1 μM). (**G**–**I**) HLA-DRA protein expression levels were detected through WB in A498 cells treated with different concentrations of elesclomol and Cu for 8, 16, and 24 h. (**J**) RNA expression levels of HLA-DRA were detected through qRT-PCR in A498 cells treated with different concentrations of elesclomol and Cu for 4, 8, 16, and 24 h. (**K**) Expression levels of HLA-DRA were measured through WB following treatment with NAC, Cu, and a combination of Cu and NAC. (**L**) Cellular ROS expression was measured under various concentrations of cuproptosis treatment (elesclomol 100 nM, Cu 5 µM and elesclomol 100 nM, Cu 10 µM). (* *p* < 0.05, ** *p* < 0.01, **** *p* < 0.0001).

The objective of this study was to investigate the potential link between HLA-DRA and cuproptosis. Prior to this, a series of in vitro experiments were conducted to verify the cytotoxicity of ccRCC cells with respect to cuproptosis. A498 cells were treated with different concentrations of elesclomol-Cu (ES-Cu) for 4 h, followed by 24 h of incubation in fresh medium to detect cell viability. ES-Cu demonstrated a significant killing effect on ccRCC cells, with the killing ability increasing as the concentration of elesclomol increased (Figure 5A). However, no significant change in the killing ability was observed when the concentration of elesclomol exceeded 100 nM (Figure 5B). This suggests that 100 nM elesclomol is the most appropriate concentration for inducing cuproptosis in ccRCC cells. Furthermore, as metal ions can also induce cell death through apoptosis, we investigated whether the cytotoxic effect observed in the previous experiments was due to apoptosis rather than cuproptosis per se. We evaluated the impact of potential apoptosis on ccRCC cells in the experiments. Cells treated with elesclomol and varying concentrations of Cu, and they exhibited a 4–9-fold increase in intracellular levels of Cu ions (Figure 5C). However, the percentage of apoptotic cells did not significantly differ from that in the control group (Figure 5D,E). These results suggest that apoptosis did not interfere with the experimental results. In conclusion, we collected data on the effects of cuproptosis on ccRCC. These results serve as a foundation for future research. 

To further investigate the relationship between cuproptosis and HLA-DRA, A498 cells were treated with elesclomol (100 nM) and Cu (1 µM), and the protein expression levels of HLA-DRA were examined. The expression level of HLA-DRA was found to be elevated compared to cells without ES + Cu treatment (Figure 5F). Based on these results, we further explored the relationship between cuproptosis and HLA-DRA by treating ccRCC cells with different concentrations of elesclomol and Cu for 8, 16, and 24 h (Figure 5G–I). These results were consistent with our finding that cuproptosis increases the expression of HLA-DRA. However, it was unclear whether this increase was due to pre- or post-transcriptional regulation, which required further validation. Therefore, we examined the levels of HLA-DRA RNA after cuproptosis treatment, as changes in RNA levels typically occur before changes in protein levels, in accordance with the central dogma. The duration of the cuproptosis treatment time was set to 4 h, 8 h, 16 h, or 24 h. Treatment of ccRCC cells with different concentrations of elesclomol and Cu resulted in a significant increase in the RNA levels of HLA-DRA compared to the control group. Furthermore, the RNA levels of HLA-DRA continued to increase with the increase in copper ion and elesclomol concentrations (Figure 5J). Therefore, we suggest that cuproptosis can further increase HLA-DRA protein levels through enhancing HLA-DRA RNA levels. Based on these findings, we investigated the mechanism by which cuproptosis controls HLA-DRA. Increasing the concentration of copper led to increased intracellular reactive oxygen species (ROS) levels (Figure 5L). This indicates that cuproptosis significantly enhances the generation of intracellular ROS. Therefore, we further explored the potential association between ROS and HLA-DRA. This study analyzed HLA-DRA expression after treating cells with copper, N-acetylcysteine (NAC; a scavenger of ROS), or a combination of copper and NAC. The results showed that scavenging ROS in the Cu + NAC group led to a decrease in HLA-DRA expression, suggesting that cuproptosis enhances ROS generation and stimulates HLA-DRA expression (Figure 5K).

### 2.6. Cuproptosis Improves the Outcomes of Anti-PD-1 Treatment In Vivo

**Figure 6:**
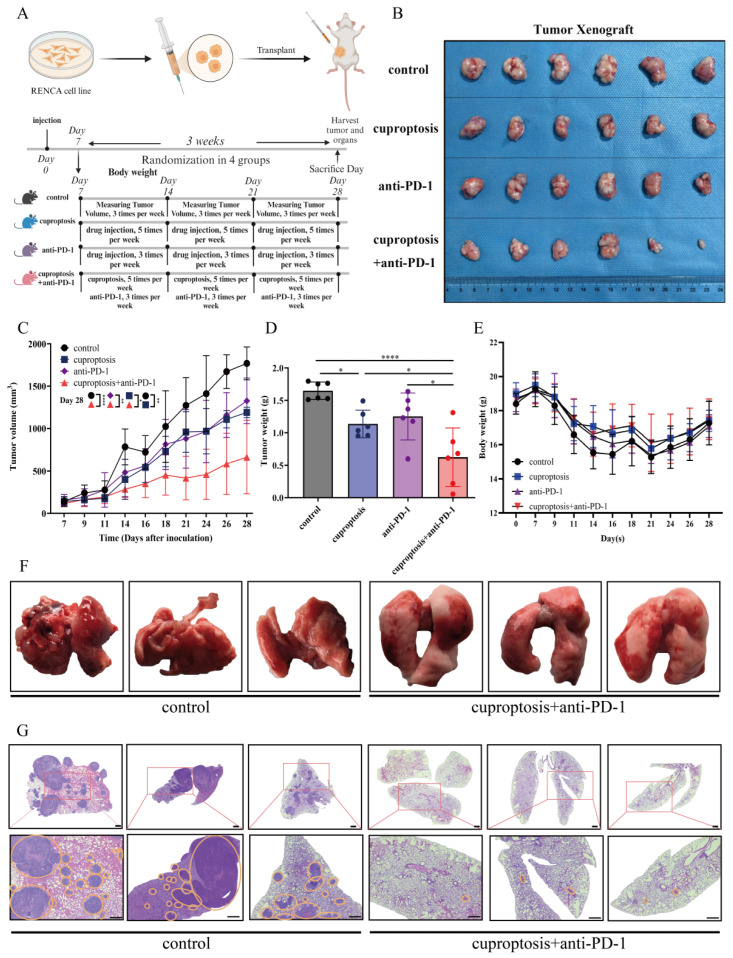
Cuproptosis improves the outcomes of anti-PD-1 treatment in vivo. (**A**) The construction of cell line-derived xenograft (CDX) models as well as cuproptosis; anti-PD-1 treatment administration schedule. (**B**) Representative images of mice subcutaneous syngeneic tumors. (**C**) The relative tumor volumes were measured 3 times per week until the sacrifice day in CDX models. (**D**) The tumor weights after the indicated treatments (cuproptosis, anti-PD-1, and cuproptosis + anti-PD-1) in CDX models. (**E**) The body weights of the mice were measured (3 times per week) throughout the experiment. (**F**) Photographs of lungs: untreated group and cuproptosis + anti-PD-1 treatment group. (**G**) H&E staining images of lungs: untreated group and cuproptosis + anti-PD-1 treatment group. Metastatic nodules are indicated by yellow circles. Scale bar: 500 μm. (* *p* < 0.05, ** *p* < 0.01, **** *p* < 0.0001).

In this study, we found that cuproptosis induces the expression of HLA-DRA and, at the same time, that the expression of HLA-DRA is strongly correlated with the sensitivity to immunotherapy in renal cancer. These findings suggest that cuproptosis could potentially enhance the sensitivity to ICI therapy. Given the complexity of immunotherapy, we designed a series of in vivo experiments to examine the effects of cuproptosis, anti-PD-1 treatment, and cuproptosis combined with anti-PD-1 treatment (Figure 6A). After three weeks of treatment, we observed that the anti-PD-1 treatment alone had limited inhibitory effects on tumor growth. Although tumor volume and tumor weight appeared to decrease to some extent, when compared with the untreated group, no statistically significant differences were observed (Figure 6B,C). However, cuproptosis therapy was able to slow dtumor growth, resulting in a significant reduction in tumor volume and weight (Figure 6D). Next, we discovered that the combination of cuproptosis and ICI treatment significantly inhibited tumor growth compared to the untreated group (Figure 6C,D). This confirms our previous hypothesis that tumor patients may benefit from the combination of cuproptosis and ICI treatment. Surprisingly, we observed significant differences in tumor growth between the anti-PD-1 treatment alone and the cuproptosis treatment alone groups, compared to the combination group (Figure 6C,D). This finding indicates that cuproptosis improves the efficacy of anti-PD-1 treatment, resulting in a synergistic therapeutic effect. Additionally, we investigated the potential adverse effects of the combination treatment on the physical state of the mice. However, the statistical results demonstrated that there was no decrease in the body weight of the mice in the combination treatment group (Figure 6E, Supplementary Figure S6), confirming the safety of the combination treatment to a certain extent.

As lung metastasis frequently occurs in patients with advanced renal cancer, we aimed to simulate the treatment of advanced renal cancer by lung metastasis modeling. The therapeutic effect of cuproptosis combined with anti-PD-1 treatment for lung metastasis of renal cancer was observed. Our results indicated that the combination treatment group had significantly reduced lung metastases compared to the untreated group (Figure 6F), as confirmed by the HE staining assay (Figure 6G). Compared to the combination treatment group, the untreated group’s lung tissues exhibited a degree of cancerous transformation. These results suggest that cuproptosis can enhance the sensitivity to anti-PD-1 treatment. Additionally, the combination of cuproptosis and ICI treatment inhibited metastatic lesions to some extent.

### 2.7. Upregulated Expression of HLA-DRA via Cuproptosis Synergy with ICI Therapy: Enhanced Aggregation of CD4^+^ and CD8^+^ T Cells Associated with Increased Levels of CCL5, CXCL9, and CXCL10

**Figure 7:**
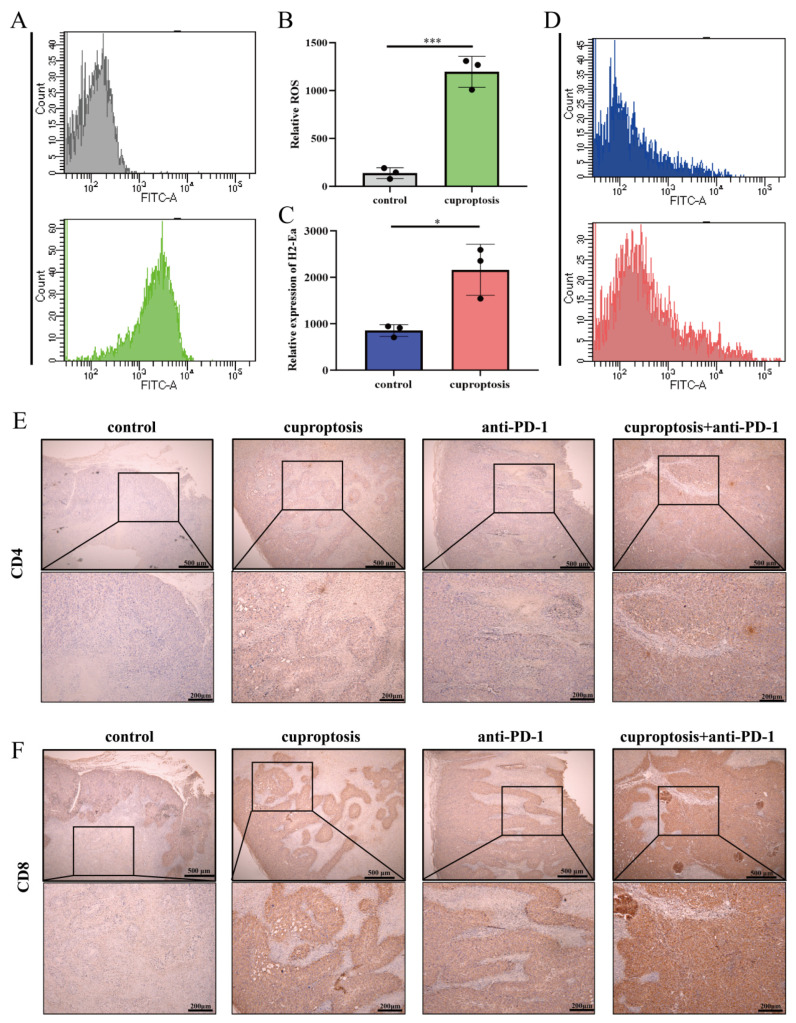
Cuproptosis improves the effectiveness of ICI therapy by promoting the aggregation of CD4^+^ and CD8^+^ T cells. (**A**,**B**) ROS levels in tumor cells were measured after cuproptosis treatment through flow cytometry detection. (**C**,**D**) H2-Ea levels in tumor cells were measured after cuproptosis treatment through flow cytometry detection. (**E**) The levels of CD4^+^T-cell infiltration in the CDX model after receiving the indicated treatments (cuproptosis, anti-PD-1, and cuproptosis + anti-PD-1) were analyzed with IHC. (**F**) The levels of CD8^+^T-cell infiltration in the CDX model after receiving the indicated treatments (cuproptosis, anti-PD-1, and cuproptosis + anti-PD-1) were analyzed with IHC. (* *p* < 0.05, *** *p* < 0.001).

Our objective was to investigate the mechanism of sensitization to ICI treatment mediated by cuproptosis. Our flow cytometry assay of tumor tissues revealed a significant increase in ROS levels after cuproptosis treatment (Figure 7A,B). We examined the mouse homolog of HLA-DRA, H2-Ea, to determine whether cuproptosis induced HLA-DRA expression. After cuproptosis treatment, the expression of H2-Ea markedly increased (Figure 7C,D), which was consistent with the results of our in vitro experiments. To explain the potential reasons for the sensitization to ICI therapy with cuproptosis treatment, we analyzed tumor tissues by serial sections and evaluated the infiltration of CD4^+^ and CD8^+^ T cells among different treatment groups using immunohistochemistry. The results showed that CD4^+^ and CD8^+^ T-cell infiltration increased in all three treatment groups compared to the untreated group (Figure 7E,F; Supplementary Figure S7A,B). Furthermore, the combined treatment group exhibited a more pronounced infiltration of CD4^+^ and CD8^+^ T cells compared to the anti-PD-1 treatment group (Figure 7E,F; Supplementary Figure S7A,B), suggesting that cuproptosis increases the expression of ts-MHC, leading to immune cell aggregation and infiltration. 

**Figure 8:**
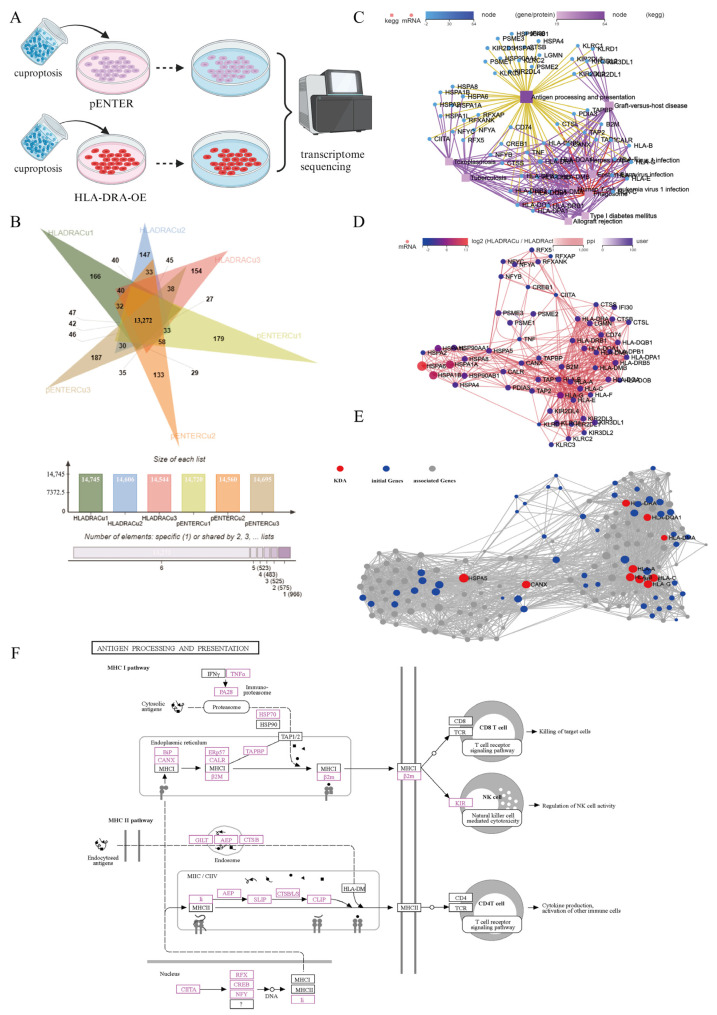
Cuproptosis modifies the immune microenvironment of tumors via HLA-DRA. (**A**) Experimental diagram that illustrates the process of transcriptome sequencing. (**B**) Venn diagram illustrating differential gene expression between six cuproptosis-treated samples (three HLA-DRA-OE-A498 samples and three pENTER-A498 samples). (**C**) The KEGG pathway relation network analysis was used to identify the biological processes that the DEGs may be involved in between HLA-DRA-OE-A498 and pENTER-A498 cells after treatment with cuproptosis. (**D**) The protein–protein interaction (PPI) network for genes enriched in the pathway relation network. (**E**) Key driver gene analysis based on the PPI network. (**F**) Annotation of differentially expressed genes in antigen processing and presentation using KEGG pathway maps.

To confirm the effect of cuproptosis on the TME through HLA-DRA, we performed bulk transcriptome sequencing of HLA-DRA-OE-A498 and pENTER-A498 cells (A498 cells transfected with the pENTER vector served as negative controls) treated with cuproptosis (Figure 8A). Correlation analysis was initially performed on all six samples to evaluate the reproducibility of biological samples within groups (Supplementary Figure S8A). Subsequently, we confirmed the reproducibility of the samples within groups, as well as the differences between samples between groups, using principal component analysis (PCA) (Supplementary Figure S8B). Based on this analysis, we compared the expression of the differential genes between the different samples and plotted a Venn diagram (Figure 8B). We conducted an analysis of differentially expressed genes (DEGs) for two sample groups. A total of 1307 DEGs were identified under the conditions of |log2FC| ≥ 1 and Q value ≤ 0.05. Of these, 771 genes were significantly upregulated and 536 genes were significantly downregulated (Supplementary Figure S8C). To clarify the potential biological functions of DEGs, a KEGG analysis was performed. The KEGG pathway relation network of the DEGs was mapped, revealing that they may be primarily involved in cellular processes, organismal systems, and human diseases. Notably, the DEGs were significantly enriched in the antigen processing and presentation pathways, which were in a central position (Figure 8C, Supplementary Figure S8D). The antigen processing and presentation pathways were further analyzed, and we found that 64 DEGs were enriched in this pathway in our dataset. To understand the co-expression relationships and interactions between the DEGs, we mapped the protein–protein interaction (PPI) network for those DEGs (Figure 8D). Key driver gene analysis (KDA) was performed based on PPI relationships, and we identified genes that were regulated by DEGs and were in a major regulatory position (Figure 8E). Finally, we labeled the identified DEGs to clarify their potential regulatory relationships with the biological processes involved. DEGs may play a role in anti-tumor immune responses through affecting the T-cell receptor signaling pathway, regulating NK cell activity and cytokine production, and activating immune cells (Figure 8F). Bulk transcriptome sequencing revealed that cuproptosis impacts the regulatory pathways responsible for processing and presenting antigens in an HLA-DRA-dependent manner. Moreover, it may improve the tumor immune microenvironment by activating T-cell receptor signaling pathways and NK cell activity. 

**Figure 9:**
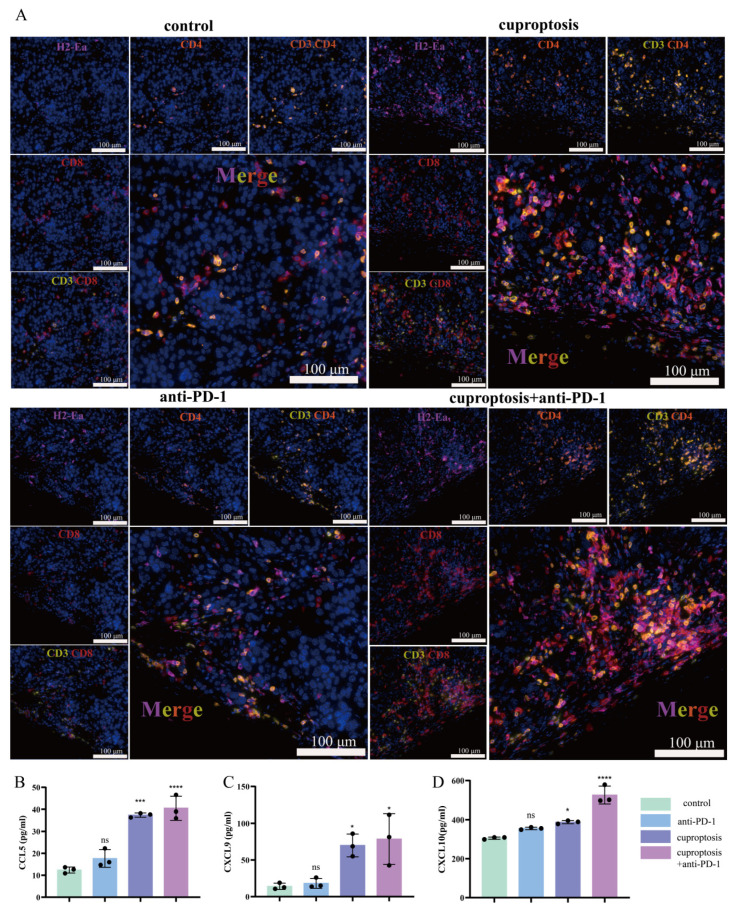
Cuproptosis increases the secretion of CCL5, CXCL9, and CXCL10 via HLA-DRA, which is presumably involved in promoting CD4^+^ and CD8^+^T cells’ aggregation in the TME. (**A**) CD3^+^CD4^+^ and CD3^+^CD8^+^ T-cell infiltration and H2-Ea expression between the indicated treatment groups. (**B**–**D**) CCL5, CXCL9, and CXCL10 expression levels were measured through ELISA after the indicated treatments. (* *p* < 0.05, *** *p* < 0.001, **** *p* < 0.0001).

To provide a clearer visualization of T-cell infiltration in the ccRCC TME, we conducted multiplex immunohistochemical staining of tumor tissues and analyzed the results. This study revealed low levels of total T(CD3^+^), CD4^+^, and CD8^+^T lymphocyte infiltration in the tumor tissues for both the untreated group and the group treated with anti-PD-1 alone. However, when the mice were treated with cuproptosis, there was a significant increase in the total number of T cells recruited in the tissues, and the infiltration of CD4^+^ and CD8^+^T cells was more pronounced. This effect was even more evident in the group that was treated with cuproptosis and anti-PD-1 (Figure 9A, Supplementary Figure S9A–D). Based on our previous transcriptome sequencing analysis results, we suggest that cuproptosis enhances the expression of ts-MHC, leading to the aggregation and infiltration of immune cells. Furthermore, we aimed to investigate the cause of the significant increase in T cells present in the TME following combination therapy. To achieve this, we measured the levels of chemokines (CCL5, CXCL9, and CXCL10) in tumor tissues using ELISA across different treatment groups. The results indicated that chemokine levels were significantly higher in the group treated with cuproptosis, as well as in the group treated with a combination of cuproptosis and anti-PD-1 (Figure 9B–D). Considering the crucial role of chemokine secretion in T-cell recruitment during the tumor immune response, we concluded that cuproptosis could activate a robust immune response through stimulating chemokine secretion. 

## 3. Discussion

ICI therapy has been widely used in the treatment of patients with advanced kidney cancer [31]. However, due to the complexity and variability of the TME and the insufficient expression of MHC molecules, the off-target effects of ICI therapy have resulted in long-term survival in only a few patients with kidney cancer [32]. Improving the therapeutic efficacy of immunotherapy is a crucial clinical issue that requires immediate attention for the treatment to benefit more patients. Cuproptosis is a recently discovered form of RCD that has garnered significant attention in the academic community [33]. It is currently believed that RCD can enhance anti-tumor immune responses [34,35], regulate the tumor immune microenvironment [36,37,38], induce immune cell aggregation [39], and improve the immunotherapeutic effects on malignant tumors [37,40]. In this study, we investigated the effects of cuproptosis on MHC-II expression and its impact on the TME. We demonstrated that cuproptosis can enhance immune cell infiltration by promoting the molecular expression of MHC-II and, in turn, sensitize the TME to ICI therapy. This study offers a new perspective on how we can explore the potential strategies for enhancing treatments with ICIs.

In this study, HLA-DRA was identified as a key CRG using multiplex analysis. Our findings suggest a strong positive correlation between HLA-DRA and the expression of the immune checkpoints CD274 (PD-L1), PDCD1 (PD-1), and CTLA4. Consequently, a detailed analysis of HLA-DRA was conducted to explore its potential contribution to ccRCC anti-tumor immunity and immunotherapy.

HLA-DRA is mainly involved in encoding the MHC-IIα chain [20]. Originally, it was widely believed that MHC-II molecules were expressed in antigen-presenting cells (APCs), and research has shown that HLA-DRA plays a crucial role as an MHC-II molecule in APC cells through various mechanisms [21,22,23]. However, recent studies have confirmed the expression of MHC-II in various tumors, including breast, melanoma, colorectal, and cervical cancers. These MHC-II tumors are collectively referred to as ts-MHC-II [24,25,26,27]. Moreover, recent research has verified that ts-MHC-II tumors have a significant impact on tumor immunity, and that their existence is associated with advantageous anti-tumor immune reactions [41]. Additionally, ts-MHC-II triggers crucial immune responses, such as the activation of CD4^+^ and CD8^+^T cells, making it a potentially valuable clinical biomarker for T-cell-inflamed tumors and for the efficacy of ICI therapy [42]. In our study, for the first time, we demonstrated the expression of HLA-DRA as a ts-MHC class II in ccRCC cell lines and clinicopathological tissues using molecular biological experiments. This expands the HLA-DRA expression profile and provides a solid theoretical foundation for our research. These results suggest that the expression of HLA-DRA as ts-MHC II in kidney cancer is a potential breakthrough. Considering the tendency of tumor cells to reduce the expression of MHC I, thereby evading the killing effect of cytotoxic T lymphocytes (CTLs), the traditional killing of tumors by CD8^+^T cells often fails to achieve the expected outcome [23,43,44]. The presence of MHC II on the tumor surface may provide new hope for tumor therapy and improve the prognosis of ccRCC patients through activating multiple immune cells. Therefore, we aimed to investigate novel approaches for enhancing HLA-DRA expression on the surface of tumor cells.

A current obstacle to preventing the in-depth study of ts-MHC-II is the incomplete understanding of how ts-MHC-II expression is regulated [45]. This study explored the relationship between cuproptosis and HLA-DRA. Notably, through a series of in vitro experiments, we demonstrated that cuproptosis can upregulate HLA-DRA protein expression. To determine whether this upregulation was caused by pre-transcriptional or post-transcriptional regulation, we analyzed the RNA levels of HLA-DRA in ccRCC cells treated with elesclomol and Cu. Our findings indicated that treatment with various concentrations of elesclomol and Cu significantly increased the RNA levels of HLA-DRA compared to the control group. ts-MHC-II, in addition to its constitutive expression, can be regulated by tumor cells through epigenetic modifications, as well as being dependent on IFN-γ induction [46,47]. To the best of our knowledge, data on the role of cuproptosis in the regulation of ts-MHC-II are lacking. The present study elucidated the possible regulatory mechanisms of ts-MHC-II, which may offer a novel approach to enhancing anti-tumor immune responses. Various nanomaterials have been developed that are capable of inducing cuproptosis [48,49], and utilizing these nanomaterials to enhance the expression of ts-MHC-II-HLA-DRA may offer a novel approach to augmenting anti-tumor immune responses. The findings of the present study provide insights that should prompt further investigations of the regulatory effects of cuproptosis on other ts-MHC-II.

Furthermore, our analysis of pathological tissues from 150 ccRCC patients yielded evidence supporting a positive correlation between HLA-DRA and PD-L1 expression. An analysis of HLA-DRA in an external database confirmed that patients with high HLA-DRA expression levels were more sensitive to ICI treatment. Given that we have demonstrated that cuproptosis increases the expression levels of HLA-DRA, we hypothesize that promoting the expression of HLA-DRA in ccRCC patients through cuproptosis will enhance the sensitivity to immune checkpoint blockade. The combination of cuproptosis and ICI therapy, therefore, has the potential to partially address the issue of a low response to ICI therapy and thus improve the patient prognosis. Of course, further in vivo experiments are needed to confirm this hypothesis.

Subsequently, we conducted animal experiments to evaluate the therapeutic effects of anti-PD-1, cuproptosis, and cuproptosis combined with anti-PD-1. Our experiments showed that the use of anti-PD-1 alone in the treatment of kidney cancer inhibited tumor growth to a certain extent, but no statistically significant differences were observed compared to the control group. Theoretically, ICI therapy could be effective in most patients. However, the available studies do not support this hypothesis [50]. The efficacy of ICI therapy varies significantly among patients, with only 10–25% of kidney cancer patients being sensitive to ICI therapy [51]. Therefore, it is not surprising that such experimental results were produced. Exploring strategies to improve the sensitivity to ICI treatment from a new perspective was also one of the important research objectives of this study. In the in vivo experiments, we observed the inhibitory effect of cuproptosis on tumor growth, which was consistent with the results of the in vitro experiments. We were surprised to find that the tumor growth rate was significantly lower when cuproptosis was combined with anti-PD-1 treatment compared to anti-PD-1 treatment alone. This confirms our previous hypothesis that cuproptosis can enhance the sensitivity to ICI treatment. At the same time, the combination treatment did not have a significant effect on the body weight of mice, which to a certain extent proves the safety of the combination treatment and provides theoretical support for its potential clinical application. Furthermore, we discovered that the combination of cuproptosis and anti-PD-1 treatment effectively suppressed the growth of metastatic tumors in the lungs. Considering that patients with advanced renal cancer frequently develop lung metastases [52], we suggest that the implementation of cuproptosis in combination with anti-PD-1 therapy may have a positive therapeutic impact.

After tumor formation, the cellular and non-cellular components within the TME interact to gradually form an immunosuppressive and tumor-promoting microenvironment. This inhibitory TME poses a significant obstacle to the success of immunotherapy [53] Therefore, remodeling of the TME is a key factor that affects ICI treatment. Our in vivo experiments demonstrated that cuproptosis increased the expression of E2-Ha—the mouse homolog of HLA-DRA, in tumor cells, and we found that the combination treatment resulted in significantly greater recruitment of CD4^+^T and CD8^+^T cells in tumor tissues when compared with anti-PD-1 treatment alone. We believe that cuproptosis enhances the expression of ts-MHC II, which, in turn, recruits more CTLs, ultimately resulting in sensitizing tumor cells to ICI treatment. To confirm that cuproptosis is mediated by HLA-DRA and, thus, affects the TME, bulk transcriptome sequencing analysis was conducted on six samples. Further functional analysis of the DEGs revealed significant enrichment in antigen processing and presentation pathways. Subsequent labeling of DEGs in the signaling pathways confirmed that cuproptosis may alter the anti-tumor orientation of the TME in an HLA-DRA-dependent manner through activating T-cell receptor signaling pathways and NK cell activity. These findings are consistent with the results of the in vivo experiments and support our hypothesis at the transcriptomic level. Several studies have shown that upregulation of ts-MHC-II, which is typically associated with increased numbers of CD4^+^ and CD8^+^ tumor-infiltrating lymphocytes, leads to tumor rejection and improves the immunotherapy response [25,39,43,45]. Our findings are consistent with this foundation. Based on this foundation, we then investigated how HLA-DRA promotes T-cell infiltration in the TME. The expression of CCL5, CXCL9, and CXCL10 was significantly elevated in the cuproptosis treatment group, as well as in the combination treatment group. These chemokines can enhance the body’s anti-tumor effects by inducing immune cell migration, which is important for the treatment of malignant tumors. For example, CCL5 binds to the CCR5 receptor on the surface of CTLs, recruiting them to the TME and, ultimately, promoting tumor regression [54]. A previous study found that CXCL9 expression enhances T-cell infiltration and suppresses tumor growth [55]. Additionally, CXCL10 secretion can induce CD8^+^T-cell homing in the TME [56]. Our bioinformatics analysis also found a significant positive correlation between HLA-DRA and the expression of CCL5, CXCL9, and CXCL10. Based on this analysis, we suggest that HLA-DRA may regulate the immune surveillance of tumors by immune cells through influencing the expression of chemokines during the cuproptosis treatment, which ultimately enhances the efficacy of ICI therapy by reducing the immunosuppressive TME.

## 4. Materials and Methods

### 4.1. Data Collection and Screening of Cuproptosis-Related Genes (CRGs)

All transcriptome data and relevant clinical information of patients were downloaded from the The Cancer Genome Atlas (TCGA) database (https://portal.gdc.cancer.gov/, accessed on 28 February 2023). The RNA-Sseq data present in the transcript data of ccRCC transcript data were downloaded to obtain gene expression information according to the sample name. [Details of how to download RNA-Seq data: After entering the website (https://portal.gdc.cancer.gov/), click ‘Projects’, ‘Program’, ‘TCGA’, and ‘TCGA-KIRC’ in order, and then click on the ‘TCGA-KIRC’ hyperlink; in the upper left corner of the pop-up dialog box, click on ‘Save New Cohort’, write the name (TCGA-KIRC), and finally click on ‘Save’. Close the pop-up window, and in the upper left corner of the drop-down box, select the project that you just saved. Click on ‘Repository’, and in turn, then select ‘Experimental Strategy-RNA-Seq’, ‘Data Category-transcriptome profiling’, ‘Data Type-Gene Expression Quantification’, ‘Workflow Type-STAR-Counts’, and ‘Access-open’, and then download the selected data]. All gene expression files were sorted using Perl software (https://www.perl.org, 28 February 2023), and the original expression levels of the genes were corrected. An expression matrix was constructed, including 541 normal samples and 72 ccRCC samples; it displayed the gene expression (where the row names of the matrix are the names of the genes and the column names are the names of the samples, showing the expression of the genes in each sample) corresponding to each sample. Subsequently, we organized the transcriptome data, downloaded the annotation information (protein-coding genes, small/long ncRNA genes, etc.) from the gencode database, using Perl to distinguish them as mRNAs or non-coding RNAs, and obtained two matrices, using the mRNA matrix for subsequent studies. The corresponding (clinical, xml format) files were also downloaded from the TGCA database.

The clinical information of patients with ccRCC was downloaded for further analysis of a series of data, including age, sex, follow-up time, survival status, and T/N/M staging. Immune responses were as predicted in TIDE (http://tide.dfci.harvard.edu/, accessed on 28 February 2023) based on immune expression data. The GEPIA (http://gepia2.cancer-pku.cn/#index, accessed on 28 February 2023) and TISDB (http://cis.hku.hk/TISIDB/, accessed on 28 February 2023) databases were used for HLA-DRA expression analysis and for immunostimulator and chemotaxis correlation analysis of chemokine factors and chemokine receptors. The effect of HLA-DRA expression levels on responsiveness to nivolumab treatment in patients with ccRCC was analyzed using the TISIDB database (http://cis.hku.hk/TISIDB/browse.php?gene=HLA-DRA, 28 February 2023).

The screening of CRGs was based on known cuproptosis genes (*NFE2L2*, *NLRP3*, *ATP7B*, *ATP7A*, *SLC31A1*, *FDX1*, *LIAS*, *LIPT1*, *LIPT2*, *DLD*, *DLAT*, *PDHA1*, *PDHB*, *MTF1*, *GLS*, *CDKN2A*, *DBT*, *GCSH*, and *DLST*); we defined cuproptosis genes as the genes that have been reported in the literature and confirmed to affect cuproptosis [19,57,58,59,60,61], while CRGs (cuproptosis-related genes) were defined as genes that may be associated with these 19 cuproptosis genes. The mRNA expression of cuproptosis genes was determined through R and its limma package; further co-expression analysis was then performed on the obtained mRNA expression levels of cuproptosis genes and the mRNA levels of other genes in the gene list of the ccRCC samples. For rigorous screening, variables with |Cor| > 0.6 and *p* < 0.001 were considered to have significant correlations. Correlation coefficients greater than zero represented a positive correlation, while correlation coefficients less than zero represented a negative correlation. Furthermore, the same method was used to extract the expression of all CRGs. The expression of CRGs in patients was combined with their clinical data downloaded from the TCGA database through R and its limma package. We excluded patients without information on survival time or status in the follow-up, so as to avoid statistical errors. Univariable Cox regression analysis was used to screen co-expressed CRGs (*p* < 0.01, HR ≠ 1). Thereafter, the “glmnet”, “survival”, “caret”, “survminer”, and “cvglmnet” packages of R software (4.4.0) were used to perform LASSO regression and cross-validation analyses to screen related genes, and we found the point with the smallest error through LASSO regression analysis. Finally, multivariable Cox regression analysis was performed to identify the optimal CRGs.

### 4.2. Functional Enrichment and Drug Sensitivity Analyses

For CRGs, “BiocManager”, “DOSE”, “clusterProfiler”, and “enrichplot” were used for Gene Ontology (GO) and Kyoto Encyclopedia of Genes and Genomes (KEGG) functional enrichment analyses, and “GSEABase” was used for gene set enrichment analysis (GSEA) of HLA-DRA (*p* < 0.05 and FDR < 0.05). The high- and low-risk groups were assessed for their responsiveness to drugs (using the “pRRophetic” package in R 4.4.0), which was determined according to the half-maximal inhibitory concentration of GDSCs. 

### 4.3. Analysis of the Immune Microenvironment and Immune Infiltration of the Tumors

Immune function correlation analysis and CRG imaging were performed using the “BiocManager”, “reshape2”, and “pheatmap” packages in R (4.4.0). Immune stromal cell infiltration and tumor purity in ccRCC were assessed using the ESTIMATE algorithm. The GEPIA database was used to analyze the correlation of CRGs with immune checkpoints (PD-1, CTLA-4, and PD-L1). In addition, the “reshape2”, “ggpubr”, and “ggExtra” packages were used to analyze the correlation between HLA-DRA and immune cell infiltration in the immune microenvironment. 

### 4.4. Cell Culture and Cell Lines

Human 786-O, OS-RC-2, A498, and HK-2 (human kidney epithelioid cells) and mouse RENCA cell lines were purchased from Procell Life Science and Technology Co., Ltd., Wuhan, China. The 786-O and RENCA cells were inoculated in an RPMI 1640 medium (Gibco, Rockville, MD, USA) enriched with 10% FBS (Biological Industries, Kibbutz Beit-Haemek, Israel) for growth. The A498, OS-RC-2, and HK-2 cells were inoculated in minimum essential medium (Gibco, Rockville, MD, USA) enriched with 10% FBS along with penicillin (100 U/mL) and streptomycin (100 mg/mL). All cells were grown at 37 °C under humidified conditions of 5% carbon dioxide. The cell lines were routinely tested to rule out mycoplasma contamination. All cell lines were identified via STR authentication to ensure accuracy.

### 4.5. RNA Isolation and Quantitative Reverse-Transcription Polymerase Chain Reaction (qRT-PCR)

Total cellular RNA was isolated with the TRIzol kit (Invitrogen, Waltham, MA, USA), and the RNA concentration was determined. Complementary DNA was synthesized from total RNA using the PrimeScript RT Reagent Kit with the gDNA Eraser (Takara Biotechnology (Dalian) Co., Ltd., Shiga, Japan). Afterwards, we quantified the transcripts via qRT-PCR with the FastStart Universal SYBR Green Master Mix (Roche) and gene-specific primers; β-actin served as the internal control. The oligonucleotide sequences for qRT-PCR were as follows: 5′-TCTGGCGGCTTGAAGAATTTG-3′ (forward) and 5′-GGTGATCGGAGTATAGTTGGAGC-3′ (reverse) for HLA-DRA; 5′-AGAGCTACGAGCTGCCTGAC-3′ (forward) and 5′-AGCACTGTGTTGGCGTACAG-3′ (reverse) for β-actin. The fold changes were calculated through relative quantification using the 2^−ΔΔCt^ method.

### 4.6. Western Blot Analysis

Protein isolation was performed after cell lysis with RIPA buffer for 30 min. The isolated proteins were quantified using the bicinchoninic acid kit. Thereafter, the proteins were subjected to 12% SDS-PAGE. Subsequently, the proteins were electrotransferred onto PVDF membranes. The membranes were blocked with 5% skimmed milk for 1 h. Afterwards, the proteins were incubated overnight with the following diluted rabbit primary antibodies: HLA-DRA antibody (A10863, ABclonal, Wuhan, China) and β-actin antibody (20536-1-AP, Proteintech, Wuhan, China). After rinsing, the membranes were incubated with secondary goat anti-rabbit immunoglobulin G (PR30011, Proteintech) for 1 h. The immuno-complexes on the membranes were visualized using the chemiluminescence fluorescent detection kit (34579, Thermo Scientific, Waltham, MA, USA).

### 4.7. Cell Viability Assay

A498 cells were counted and transferred to 96-well plates at a density of 2000 cells/well. The cell viability assay was started on day 0 after 24 h of incubation. The assay was performed using the CCK-8 Kit (B34302, Selleck, Shanghai, China) according to the manufacturer’s instructions.

### 4.8. Cuproptosis Detection

A498 cells were treated with 50, 100, 150, and 200 nM elesclomol-Cu (ES-Cu) (1:1 ratio) (MCE, HY-12040) and grown in fresh media. The cell viability assay was started after 24 h of incubation. A498 cells were treated with elesclomol (100 nM) and Cu (1 μM, 5 μM), followed by digestion with trypsin. The cells were subsequently washed twice with PBS and incubated with Annexin V dye labeled with a fluorescent agent for 15 min at room temperature. A nucleic acid dye was then added and incubated for 5 min at room temperature. Finally, flow cytometry (FCM) analysis was carried out with a flow cytometer from BD Biosciences. In exploring the relationship between cuproptosis and HLA-DRA, we treated A498 cells with elesclomol (100 nM, 200 nM) and Cu (1 μM, 5 μM) for 4, 8, 16, and 24 h. Subsequently, the protein levels or RNA levels of HLA-DRA were examined. 

### 4.9. Transcriptome Sequencing Analysis

To investigate the possible effects of cuproptosis and HLA-DRA on the tumor immune microenvironment, we obtained plasmid-transfected HLA-DRA-OE-A498 cells after treatment with elesclomol and Cu for 24 h. The cells were washed three times with PBS and then collected and subjected to transcriptome sequencing analysis (BGI TechSolutions Co., Ltd., Shenzhen, China). Total cellular RNA was isolated with the TRIzol kit (Invitrogen, Carlsbad, CA, USA), and the RNA concentration was determined. After quality control according to sample and product requirements, mRNA libraries were prepared, and the processed DNA nanoballs containing multiple copies were subsequently sequenced through combinatorial probe–anchor synthesis using a high-intensity DNA nanochip technique. 

After obtaining the transcriptome data [the raw transcriptome data have been deposited in the Genome Sequence Archive of the National Genomics Data Center, China National Center for Bioinformation/Beijing Institute of Genomics, Chinese Academy of Sciences (GSA-Human: HRA007421), and they are publicly accessible at https://ngdc.cncb.ac.cn/gsa-human, 16 May 2024], the data were analyzed for Pearson’s correlation, PCA, and differential expression of DEGs, and the DEGs were analyzed for GSEA, KEGG pathway networks, PPI network diagrams, and KDA using the official software packages.

### 4.10. Mouse Syngeneic Tumor Experiment

Animal experiments were performed in accordance with the National Institutes of Health guidelines and approved by the Animal Ethics Committee of Harbin Medical University. Initially, 6–8-week-old female BALB/c mice were purchased from Liaoning Changsheng Biotechnology Co., Ltd. (Shenyang, China). After resuspension of 4 × 10^6^ RENCA cells with PBS, they were injected subcutaneously into the body of each mouse, and when the tumor volume reached ~100 mm^3^ (~7 days), the mice were randomly divided into 4 groups. Subsequently, the mice were injected intraperitoneally with elesclomol (40 mg/kg) + CuCl_2_ (0.06 mg/kg) and anti-mouse PD-1 (clone RMP1-14, 10 mg/kg). The mice were then injected with elesclomol + CuCl_2_ five times per week for three weeks, as well as with anti-mouse PD-1 three times per week for three weeks. The tumor volume was monitored during the treatment period using a Vernier caliper, with tumor volume (mm^3^) = 0.5 × length × width^2^. The mice were then euthanatized, and the transplanted tumors were photographed after 3 weeks of continuous treatment. Flow cytometry was performed on the collected tumor tissues to detect ROS levels and H2-Ea expression. The expression levels of CCL5, CXCL9, and CXCL10 in the tumor tissues were measured through ELISA. This study was approved by the Institutional Animal Ethics Committee of the First Affiliated Hospital of Harbin Medical University. Animal housing and experiments were conducted in strict accordance with the Institutional Guidelines for Care and Use.

### 4.11. Flow Cytometry Detection

Mouse tumor tissues were processed into single-cell suspensions and rinsed three times with PBS, before being incubated with DCFH-DA (HY-D0940, MCE, Princeton, NJ, USA) for 30 min at room temperature to detect ROS levels, and with FITC-anti-H2-Ea (ab93561, Abcam, Boston, MA, USA) for 30 min to detect H2-Ea levels. Finally, flow cytometry (FCM) analysis was performed with a flow cytometer (BD Biosciences, LSRFortessa, Franklin Lakes, NJ, USA).

### 4.12. Immunohistochemical (IHC) Staining and Semi-Quantitative Scoring

Tumor tissues of 150 patients with ccRCC and 30 pairs of adjacent normal tissues were supplied by Shanghai Outdo Biotech Company (HKidE180Su02). IHC staining was performed on the above-mentioned sections according to standardized procedures. The primary antibody used was anti-HLA-DRA (1:200 dilution, Rat. 17,221–1-AP, Proteintech). Staining was visualized with DAB and hematoxylin counterstaining. For semi-quantitative analysis, two pathologists independently evaluated the stained sections according to evaluation standards on a 12-point scale by calculating the immunoreactivity score [62]. For IHC staining of mice tumor tissues following standardized procedures, the primary antibodies used were anti-CD4 (67786-1-lg, Proteintech) and anti-CD8 (29896-1-AP, Proteintech). Tumor tissue was stained using a multiplex immunohistochemical kit (abs50030, absin) according to the manufacturer’s instructions. The primary antibodies used were anti-CD3 (1:200 dilution, 78588, CST), anti-CD4 (1:500 dilution, ab183685, Abcam, Cambridge, UK), anti-CD8 (1:200 dilution, ab217344, Abcam), and anti-H2-Ea (1:200 dilution, 862855, CST).

### 4.13. Statistical Analysis

Statistical analyses in the present study were performed using the R software (4.1.1) (R Foundation for Statistical Computing, Vienna, Austria) and GraphPad Prism 8.0.2 (GraphPad Software Inc., San Diego, CA, USA). The data are presented as the mean ± standard deviation. The log-rank test and the Kaplan–Meier approach were applied to determine OS. The linear correlations between two variables were established via Spearman’s correlation analysis. Student’s *t*-test was used to determine two-group differences, whilst one-way analysis of variance was used for multi-group differences. *p* < 0.05 was considered statistically significant. 

## 5. Conclusions

In summary, this study proposes a new idea for immunotherapy of ccRCC from the perspective of cuproptosis. Our findings confirm the regulatory effect of cuproptosis on HLA-DRA, highlighting the expression of HLA-DRA as a ts-MHC-II and its positive impact on ICI treatment. Notably, our findings demonstrate that cuproptosis promotes the recruitment of CD4^+^ and CD8^+^T-lymphocyte cells in the TME by upregulating HLA-DRA expression, thereby enhancing the responsiveness to ICI therapy. This research provides new insights into improving anti-tumor immunotherapy and confirms the potential of cuproptosis as a new modality for cancer therapy.

## Data Availability

Data are contained within the article.

## Supplementary Materials

The following supporting information can be downloaded at https://www.mdpi.com/article/10.3390/ph17060678/s1, Figure S1: Sankey diagram of CRGs and cuproptosis-related mRNAs in ccRCC. Figure S2: GO and KEGG enrichment analyses of CRGs differentially expressed in the two groups: (A,B) GO enrichment analysis of CRGs. (C,D) KEGG enrichment analysis of CRGs. Figure S3: Co-expression of CRGs with three immune checkpoints (PDCD1, CTLA-4, and CD274): (A) Co-expression of FOSL1 with three immune checkpoints. (B) Co-expression of MOCS2 with three immune checkpoints. (C) Co-expression of PDPN with three immune checkpoints. (D) Co-expression of TRAIP with three immune checkpoints. (E) Co-expression of TRIP13 with three immune checkpoints. Figure S4: GSEA analysis of HLA-DRA in ccRCC. Figure S5: (A,B) Analysis of HLA-DRA and immune cell infiltration in the tumor immune microenvironment. Figure S6: Statistical analysis of mice’s weight in each group at the termination of the experiment (day 28). Figure S7: IHC scoring of CD4 and CD8 expression in different treatment groups: (A) IHC scoring of CD4 expression in different treatment groups. (B) IHC scoring of CD8 expression in different treatment groups. (The staining intensity was graded independently three times in all cases.) Figure S8: Analysis for transcriptome sequencing: (A) Correlation analysis between six samples. (B) PCA analysis between different treatment groups. (C) DEGs in two different groups. (D) GSEA enrichment analysis for DEGs. Figure S9: CD3^+^T-cell infiltration between different treatment groups: (A) Untreated group. (B) Anti-PD-1 treatment group. (C) Cuproptosis treatment group. (D) Combined treatment group. Table S1. Points in the Figure 3A scatter plot represent the expression difference of HLA-DRA between responders and non-responders in various data sets.
