# Supplementary material for: Improved Immunotherapy Outcomes via Cuproptosis Upregulation of HLA-DRA Expression: Promoting the Aggregation of CD4+ and CD8+T Lymphocytes in Clear Cell Renal Cell Carcinoma
Source: Pharmaceuticals (Basel). 2024 May 24;17(6):678. doi: 10.3390/ph17060678 (PMC11206763; doi:10.3390/ph17060678)
Supplement: Supplementary file 1 [file pharmaceuticals-17-00678-s001.zip › English-Editing-Certificate.pdf]

We certify that the following article

Cuproptosis improves immunotherapy outcomes by upregulating HLA-DRA expression which promotes the aggregation of CD4+ and CD8+T lymphocytes in clear cell renal cell carcinoma

Bowen Wang, Yiwen Liu, Feng Xiong, Chunyang Wang *

has undergone English language editing by MDPI. The text has been checked for correct use of grammar and common technical terms, and edited to a level suitable for reporting research in a scholarly journal.
MDPI uses experienced, native English speaking editors. Full details of the editing service can be found at
► https://www.mdpi.com/authors/english.

MDPI Multidisciplinary Digital Publishing Institute
Basel, Switzerland
May 2024

AUTHORservices
english-80534
