# Supplementary material for: Improved Immunotherapy Outcomes via Cuproptosis Upregulation of HLA-DRA Expression: Promoting the Aggregation of CD4+ and CD8+T Lymphocytes in Clear Cell Renal Cell Carcinoma
Source: Pharmaceuticals (Basel). 2024 May 24;17(6):678. doi: 10.3390/ph17060678 (PMC11206763; doi:10.3390/ph17060678)
Supplement: Supplementary file 1 [file pharmaceuticals-17-00678-s001.zip › Table S1.pdf]

Table S1. Points in the Figure 3A scatter plot represent the expression difference of HLA-DRA between responders and non-responders in various data sets.

No	PMID	Cancer type	Group	Drug	The Number of responders	The Number of Non-responders
1	29301960	Clear cell renal cell carcinoma (ccRCC)	all	Anti-PD-1 (nivolumab)	4	8
2	29301960	Clear cell renal cell carcinoma (ccRCC)	VEGFRi	Anti-PD-1 (nivolumab)	2	0
3	29301960	Clear cell renal cell carcinoma (ccRCC)	non-VEGFRi	Anti-PD-1 (nivolumab)	2	8
