# Supplementary figures and images for: Improved Immunotherapy Outcomes via Cuproptosis Upregulation of HLA-DRA Expression: Promoting the Aggregation of CD4+ and CD8+T Lymphocytes in Clear Cell Renal Cell Carcinoma
Source: Pharmaceuticals (Basel). 2024 May 24;17(6):678. doi: 10.3390/ph17060678 (PMC11206763; doi:10.3390/ph17060678)

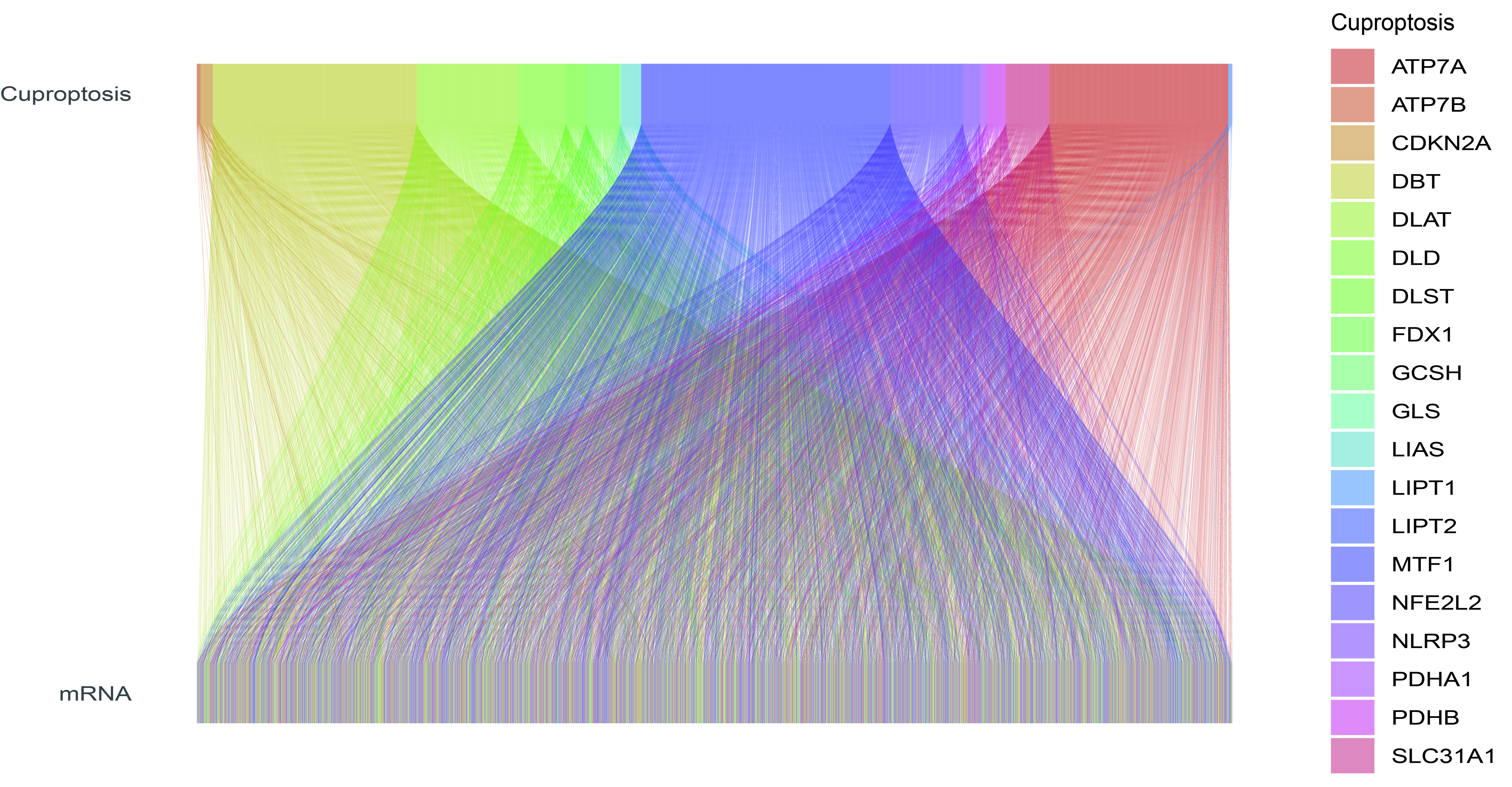

Supplement: Supplementary file 1 [file pharmaceuticals-17-00678-s001.zip › FigureS1.jpg]

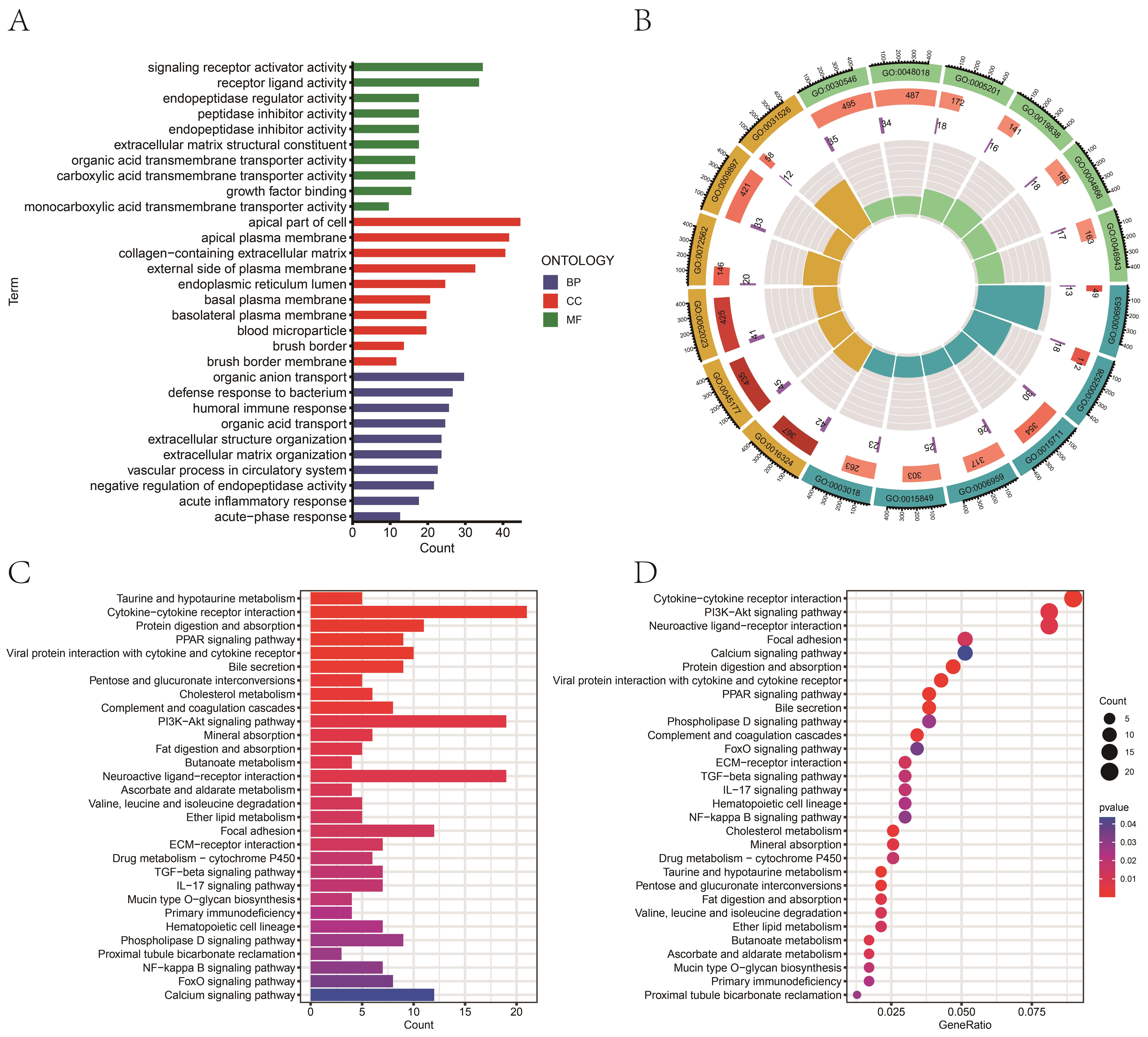

Supplement: Supplementary file 1 [file pharmaceuticals-17-00678-s001.zip › FigureS2.jpg]

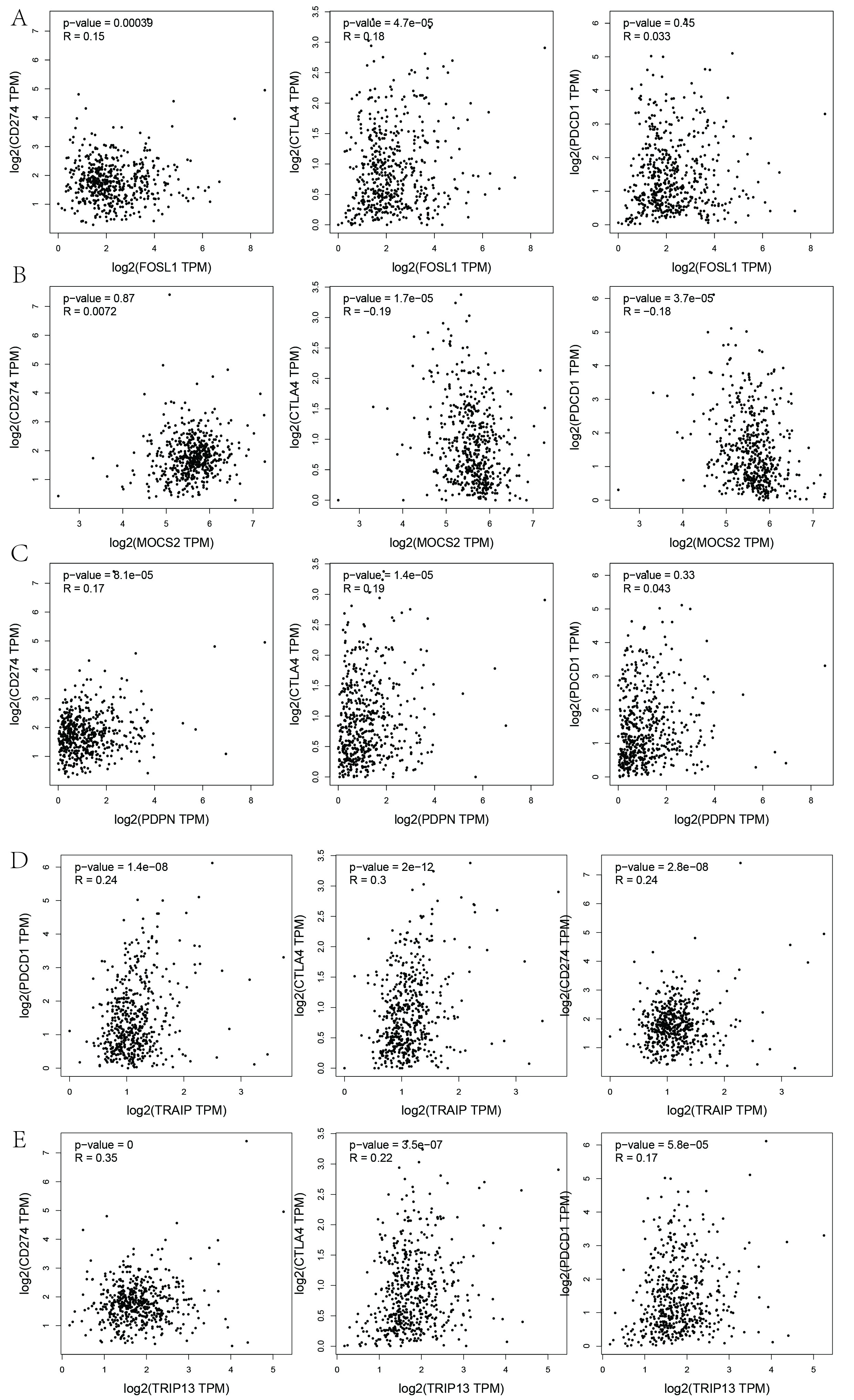

Supplement: Supplementary file 1 [file pharmaceuticals-17-00678-s001.zip › FigureS3.jpg]

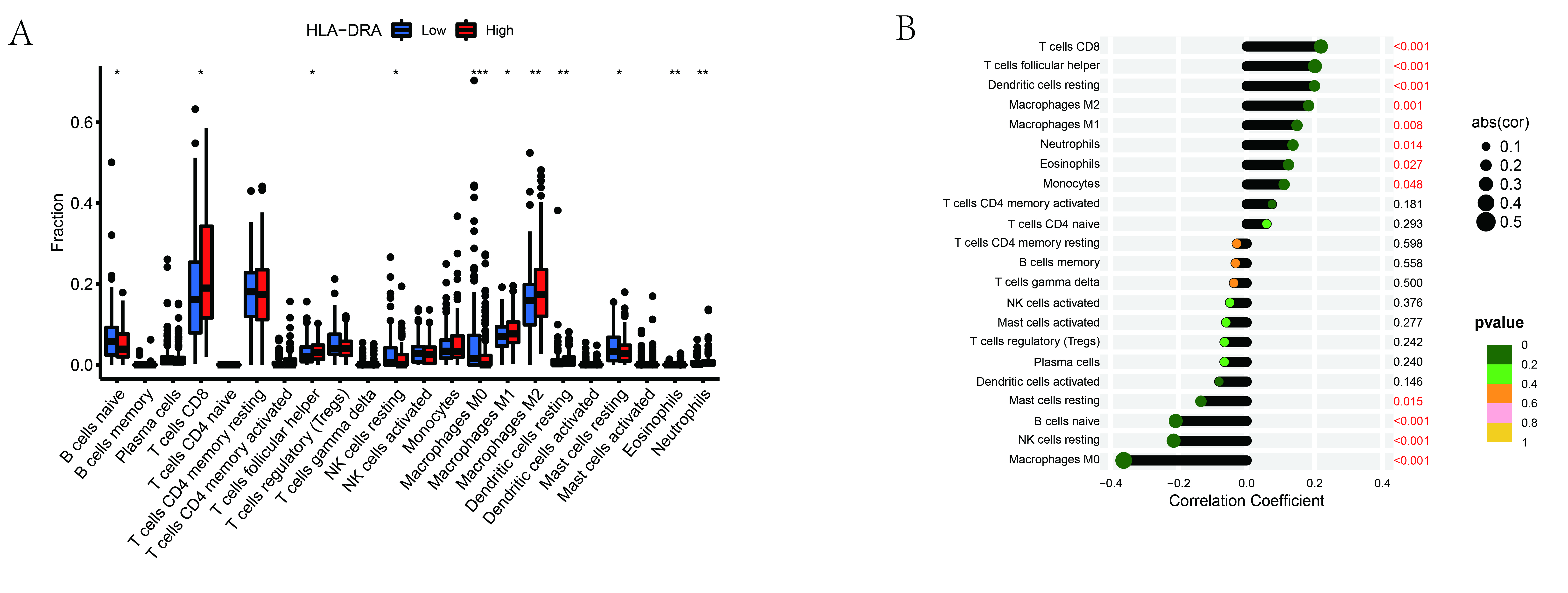

Supplement: Supplementary file 1 [file pharmaceuticals-17-00678-s001.zip › FigureS5.jpg]

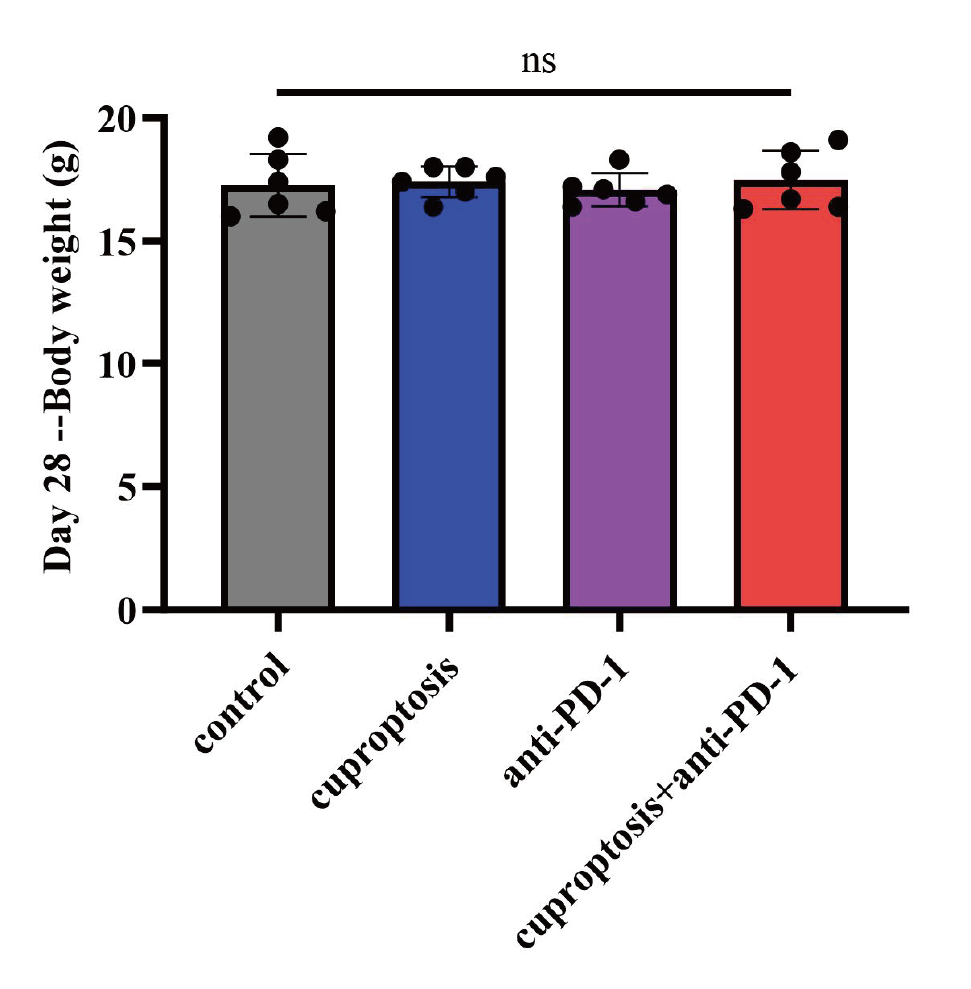

Supplement: Supplementary file 1 [file pharmaceuticals-17-00678-s001.zip › FigureS6.png]

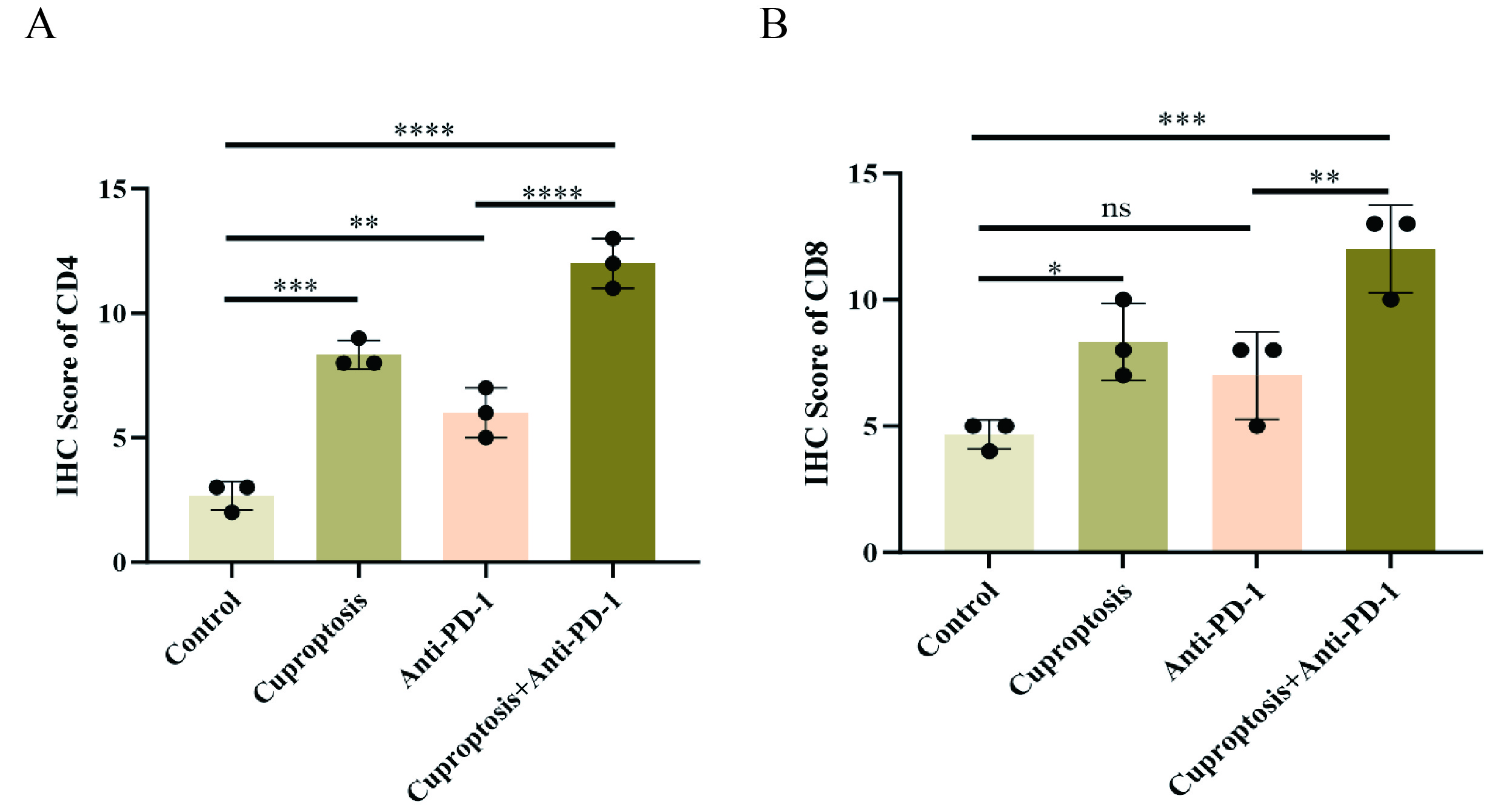

Supplement: Supplementary file 1 [file pharmaceuticals-17-00678-s001.zip › FigureS7.jpg]

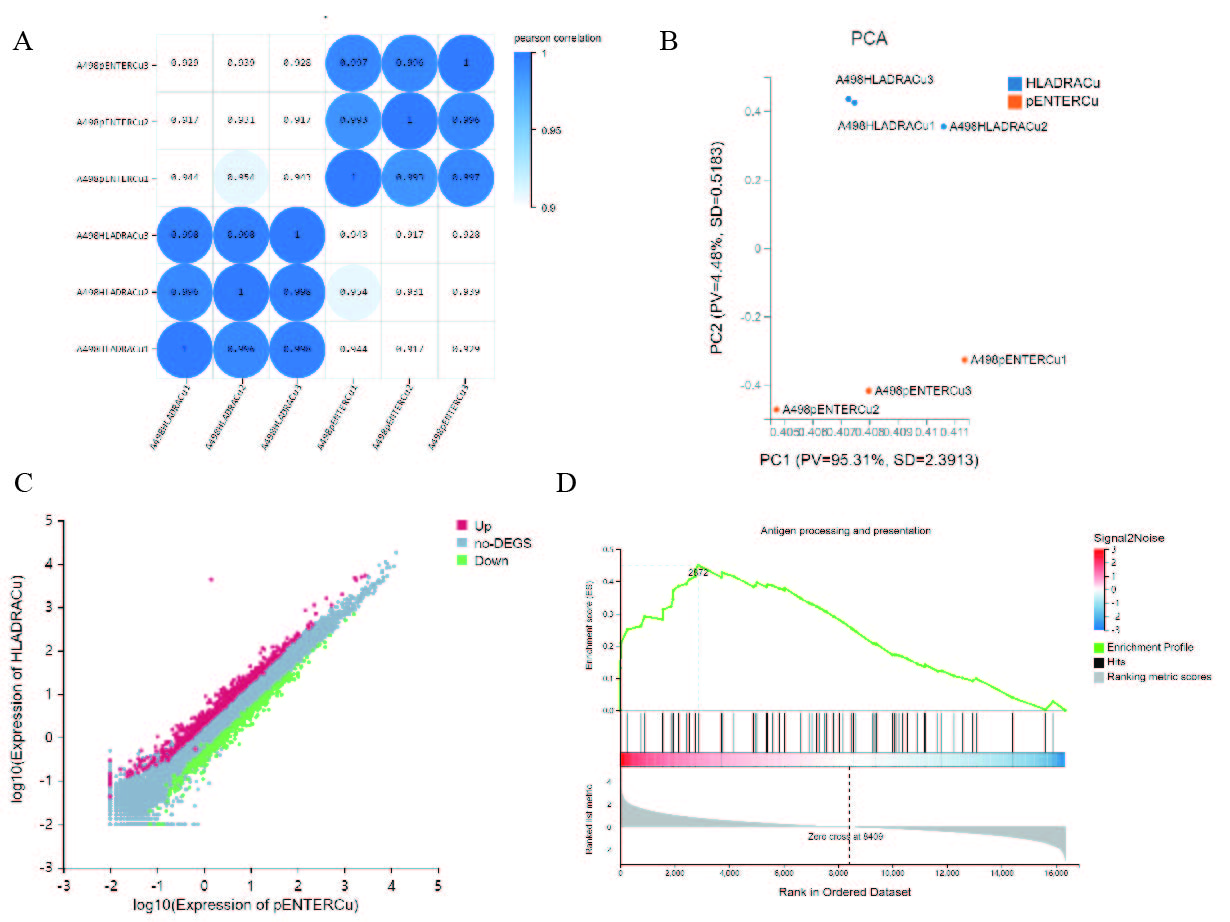

Supplement: Supplementary file 1 [file pharmaceuticals-17-00678-s001.zip › FigureS8.jpg]

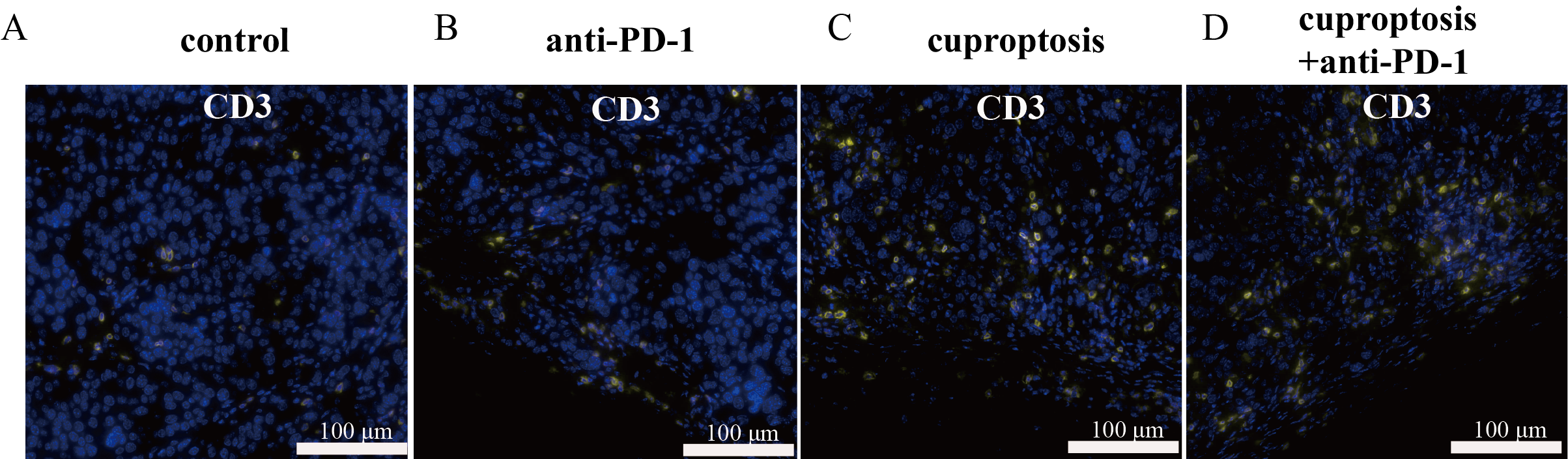

Supplement: Supplementary file 1 [file pharmaceuticals-17-00678-s001.zip › FigureS9.png]
